# Retention of antibiotic activity against resistant bacteria harbouring aminoglycoside-*N*-acetyltransferase enzyme by adjuvants: a combination of in-silico and in-vitro study

**DOI:** 10.1038/s41598-020-76355-0

**Published:** 2020-11-09

**Authors:** Shamim Ahmed, Sabrina Amita Sony, Md. Belal Chowdhury, Md. Mahib Ullah, Shatabdi Paul, Tanvir Hossain

**Affiliations:** Department of Biochemistry and Molecular Biology, School of Life Sciences, Shahjalal University of Science and Technology, Sylhet, 3114 Bangladesh

**Keywords:** Antibiotics, Antimicrobial resistance, Virtual drug screening

## Abstract

Interference with antibiotic activity and its inactivation by bacterial modifying enzymes is a prevailing mode of bacterial resistance to antibiotics. Aminoglycoside antibiotics become inactivated by aminoglycoside-6′-N-acetyltransferase-Ib [AAC(6′)-Ib] of gram-negative bacteria which transfers an acetyl group from acetyl-CoA to the antibiotic. The aim of the study was to disrupt the enzymatic activity of AAC(6′)-Ib by adjuvants and restore aminoglycoside activity as a result. The binding affinities of several vitamins and chemical compounds with AAC(6′)-Ib of *Escherichia coli*, *Klebsiella pneumoniae*, and *Shigella sonnei* were determined by molecular docking method to screen potential adjuvants. Adjuvants having higher binding affinity with target enzymes were further analyzed in-vitro to assess their impact on bacterial growth and bacterial modifying enzyme AAC(6′)-Ib activity. Four compounds—zinc pyrithione (ZnPT), vitamin D, vitamin E and vitamin K-exhibited higher binding affinity to AAC(6′)-Ib than the enzyme’s natural substrate acetyl-CoA. Combination of each of these adjuvants with three aminoglycoside antibiotics—amikacin, gentamicin and kanamycin—were found to significantly increase the antibacterial activity against the selected bacterial species as well as hampering the activity of AAC(6′)-Ib. The selection process of adjuvants and the use of those in combination with aminoglycoside antibiotics promises to be a novel area in overcoming bacterial resistance.

## Introduction

The discovery of antibiotics has undoubtedly saved millions of lives and contributed greatly to the extension of human lifespan^1^. But in recent decades an alarming threat to global public health known as antibiotic resistance (AR) or antimicrobial resistance (AMR) has manifested itself. Day by day, bacteria are evolving to escape antibiotic action and emerging as multi-drug resistant (MDR) strains to become resistant against a wide array of available antibiotics^2,3^. Antimicrobial-resistant infections are responsible for more than 50,000 deaths each year across the US and Europe only, along with thousands others worldwide^4^. The forthcoming disaster calls for an immediate action by the researchers to tackle the severity of AMR.

Bacteria become resistant to antibiotics by acquiring resistance genes by natural selection which is also accelerated by inappropriate human intervention^5,6^. The factors that drive the antibiotic resistance include mismanagement and excessive use of antibiotics in humans, and its extensive use in agriculture and livestock animals, etc^1^. As the development of new antibiotics take decades or longer, researchers are now focusing on new approaches for tackling bacterial resistance mechanisms instead. One such approach is antibiotic combination therapy which has been employed nowadays to increase the efficiency of the existing antibiotics^7–9^. Antibiotic combination therapy can be achieved by either combining two or more antibiotics or combining an antibiotic with an adjuvant molecule^10^. The utilization of various antibiotic agents at a same time in the form of combination antibiotic (antibiotic-antibiotic) and hybrid antibiotic (antibiotic-linker-antibiotic) may constrain the advancement of resistance in-vitro and sometimes in-vivo but are also accompanied with some drawbacks including increased expense, increased risk of adverse effect resulting from the destruction of the gut flora, antagonism and super infection^11^. Counting the positives and negatives, antibiotic-adjuvant approach is probably one of the most successful therapeutic strategies against antibiotic resistance at the present time^12^ and has been successfully implemented to break the resistance of several antibiotics including Amoxicillin-Clavulanic acid, Rifampin-Colistin, Tobramycin-Nitric Oxide, Piperacillin-Tazobactam, to name a few^13–15^.

Aminoglycoside antibiotics (AGAs) are a large family of antibiotics and is considered to be a member of the ‘Big Four’ classes of antibiotics (β-lactams, tetracyclines, macrolides, and aminoglycosides)^16^. Some members of AGAs are amikacin, gentamicin, kanamycin, neomycin, and dibekacin. Aminoglycosides inhibit bacterial protein synthesis by binding reversibly with high affinity to the 16S ribosomal RNA of the 30S ribosome^17^. This interaction with the rRNA induces codon misreading thus results in the mistranslation of proteins. This results in the production of polypeptides containing incorrect amino acids which damage the bacterial cell membrane^18,19^. Bacterial resistance to aminoglycosides results from some combination of three mechanisms including transport alterations, ribosomal alterations, and enzymatic modifications^17^. Inactivation of aminoglycosides by aminoglycoside modifying enzymes (AMEs) is the most common and clinically significant bacterial resistance mechanism to aminoglycosides. The aminoglycoside *N*-acetyltransferases (AACs) comprise the largest group of AMEs^19^ and is present in over 70% of gram-negative clinical isolates^20^. Few recent studies found out about the presence of *E. coli* isolates in environmental samples, chicken meat and them being resistant to most of the aminoglycosides since they exhibit AAC(6′)-Ib^21,22^. Alike *E. coli*, *Klebsiella pneumoniae* and *Shigella sonnei* have also been found to be aminoglycoside resistant having AAC(6′)-Ib^23,24^. Recent studies have reported the presence of aminoglycoside resistant strains such as *E. coli* in environmental samples^21,22^, *K. pneumoniae*^24^*,* and *S. sonnei*^23^ which are resistant to most of the aminoglycosides and indicates AAC(6′)-Ib for the resistance. AAC(6′)-Ib acetylates amino group of the aminoglycoside located at 6′ position and generates 6′-*N*-acetyl-aminoglycoside and CoA (Coenzyme A). The enzymatically modified aminoglycoside lacks bactericidal activity since it binds poorly to ribosomes and results in high levels of resistance^25^.

**Figure 1:**
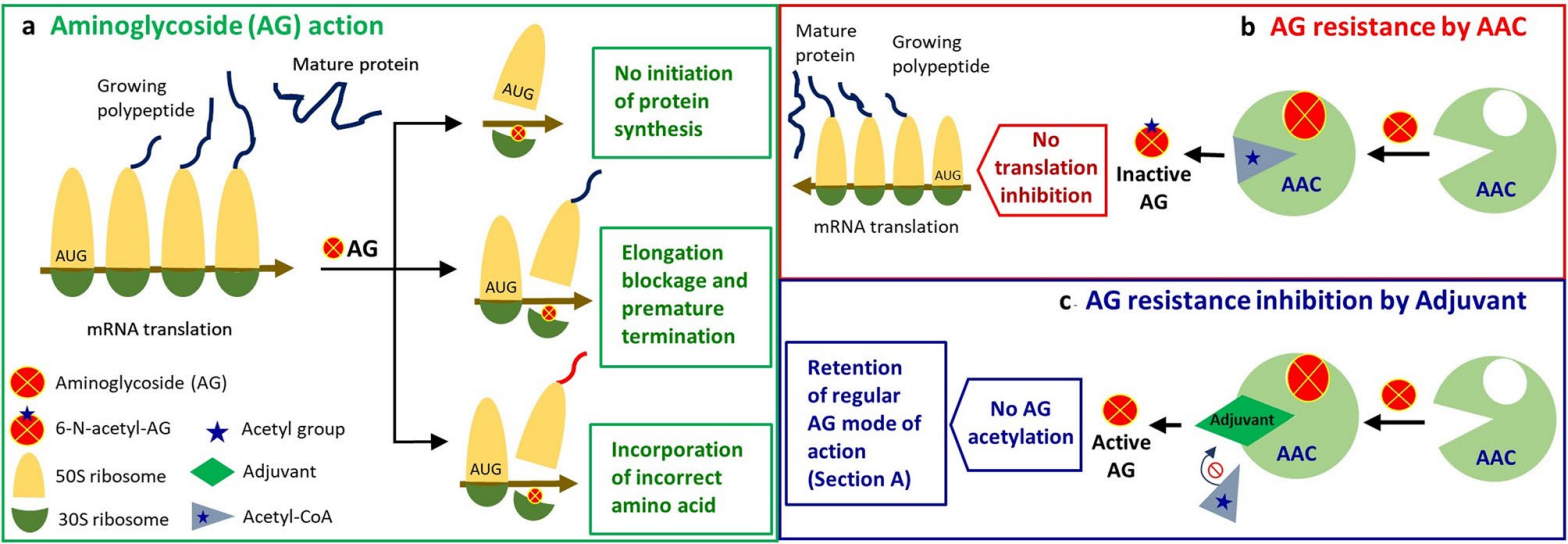
Mechanism of aminoglycoside activation, inactivation, and the provability of activity retention. (**a**) Aminoglycoside confers its bactericidal action by interfering with bacterial mRNA translation upon binding with the 30S ribosomal subunit leading to partial or complete disruption of protein synthesis^27^. (**b**) Bacteria overcomes aminoglycoside action by producing aminoglycosides modifying enzyme AAC(6′)-Ib which acetylates aminoglycoside by transferring an acetyl group donated by acetyl-CoA to generate 6′-*N*-aminoglycoside; lacking the ability to bind 30S ribosome thus rendering the antibiotic ineffective. (**c**) Adjuvant interferes with the acetyl-CoA binding site of AAC; preventing the attachment of acetyl group donor to the enzyme active site, consequently intercepting/blocking the enzymatic modification of aminoglycoside and warranting the execution of aminoglycoside action.

To retain the antibacterial property of aminoglycoside antibiotics, we postulate that blocking the action of AAC(6′)-Ib to prevent antibiotic acetylation can be an effective approach (Fig. 1). Small molecular entities, known as adjuvants, can play significant roles in interfering with the aminoglycoside modifying enzyme AAC(6′)-Ib. An adjuvant may possess very weak or no antibacterial activity on its own but can either obstruct to antibiotic resistance or accelerate antibiotic action. Adjuvant molecule inhibits bacterial resistance in several ways including inhibition of antibiotic or drug target modification, inhibition of efflux pump and, enhancement of antibiotic uptake etc^26^. But the availability of a large variety of chemical compounds that act as adjuvants such as natural compounds, organic and inorganic salts and micronutrients, makes it extremely complex to identify a potent adjuvant molecule by in-vitro experiments. Therefore, it has now become imperative that development of a systematic framework for the efficient selection of adjuvants is warranted.

Structure-based drug discovery (SBBD), a type of computer-aided drug discovery (CAAD), eases the exploration of novel compounds as potential drugs or adjuvant^28^. Nowadays, CAAD has been an integral part of drug discovery and development and it saves a lot of time and money involved in drug discovery^29^. Potential adjuvants of the aminoglycoside antibiotics can also be identified by a computational method known as molecular docking (MD) that we report in this study for the first time. MD is an SBBD method that computationally predicts the binding affinity between a macromolecule and its ligand by utilizing the structural information of a target molecule^30^. By determining the binding affinity between adjuvants and bacterial target enzyme AAC(6′)-Ib, potential adjuvants acting as AAC(6′)-Ib inhibitor can be screened.

In this study, our aim was to identify new adjuvants capable of retaining the antibacterial activity of aminoglycoside antibiotics. By in-silico molecular docking (MD), four compounds including zinc pyrithione (ZnPT), vitamin D, vitamin E, and vitamin K were identified as potential inhibitors of AAC(6′)-Ib. These compounds displayed a higher binding affinity against AAC(6′)-Ib of *E. coli, K. pneumoniae,* and *S. sonnei* than the enzyme’s natural substrate acetyl-CoA. Combination of each of these four compounds with each three aminoglycoside antibiotics including amikacin, gentamicin, and kanamycin increased each antibiotic’s action against *E. coli, K. pneumoniae,* and *S. sonnei*. Each of these compounds was also found to have an inhibitory impact on the acetylation of aminoglycosides by AAC(6′)-Ib. To the best of our knowledge, this is the first report so far that has accommodated a multitude of bioinformatics tools and structure-based molecular docking to identify adjuvants for antibiotics and which were later substantiated by subsequent laboratory experiments.

## Results

### Selection of adjuvants based on molecular docking score followed by ADMET and QSAR profiling

Instead of a conventional random selection of adjuvants, we performed Molecular Docking (MD) in terms of binding affinities of 38 chemical compounds with the enzyme for screening effective adjuvants. The highest binding affinities of AAC(6*′*)-Ib of *E. coli* with its natural substrate acetyl-CoA and all the candidate adjuvants are listed in Table 1. MD also showed several numbers of binding mode with corresponding docking score against AAC(6*′*)-Ib of studied bacteria (Supplementary Table S2). The affinity score of acetyl-CoA with the target enzyme is found to be − 6.7 kcal/mol. Vitamin K, vitamin E, ZnPT, and vitamin D scored higher than acetyl-CoA and ended up producing − 8.2, − 8.0, − 7.9 and − 6.8 kcal/mol respectively. Compared to acetyl-CoA, it is evident that the affinities of ZnPT, vitamin D, E, and K towards the enzyme are more energetically favorable owing to their high binding affinity value*.* Except ZnPT, vitamin D, E and K, other compounds listed in Table 1 scored lower than acetyl-CoA and excluded for further analysis.

**Table 1:** Docking score and inhibition constant of chemical compounds against AAC(6′)-Ib of *E. coli.*

Compound	Affinity, ΔG (kcal/mol)	Inhibition constant, K_i_ (µM)	Compound	Affinity, ΔG (kcal/mol)	Inhibition constant, K_i_ (µM)
Acetyl-CoA	− 6.7	12.12	Chromium sulfate	− 3.9	1374.38
Vitamin K	− 8.2	0.96	Sodium bicarbonate	− 3.6	2281.67
Vitamin E	− 8.0	1.35	Silver nitrate	− 3.3	3787.90
Zinc pyrithione	− 7.9	1.60	Cupric acetate	− 3.2	4485.18
Vitamin D	− 6.8	10.23	Zinc acetate	− 3.2	4485.18
Vitamin B_2_	− 5.7	65.65	Calcium carbonate	− 3.1	5310.82
Vitamin B_6_	− 5.6	77.73	Calcium chloride	− 2.3	20,521.87
Vitamin C	− 5.5	92.04	Cupric chloride	− 2.3	20,521.87
Cadmium Acetate	− 5.3	129.05	Manganese chloride	− 2.3	20,521.87
Cobalt Nitrate	− 5.3	129.05	Zinc chloride	− 2.3	20,521.87
Vitamin B	− 5.2	152.81	Magnesium chloride	− 2.3	20,521.87
Vitamin B_5_	− 5.1	180.94	Ferrous chloride	− 2.3	20,521.87
Vitamin B_3_	− 5.0	214.24	Cobalt bromide	− 2.2	24,299.58
Vitamin A	− 4.6	421.15	Cadmium iodide	− 2.0	34,069.24
Ferrous sulfate	− 4.0	1160.71	Silver chloride	− 1.2	131,648.98
Cadmium sulfate	− 4.0	1160.71	Zinc bromide	− 1.2	131,648.98
Copper sulfate	− 4.0	1160.71	Magnesium carbonate	− 1.1	155,883.22
Manganese sulfate	− 4.0	1160.71	Calcium sulfate	− 1.1	155,883.22
Zinc sulfate	− 4.0	1160.71	Magnesium sulfate	− 1.1	155,883.22

Assessment of absorption, distribution, metabolism and toxicity (ADMET) profiling were done and found our selected adjuvants have no barrier to be used with antibiotics (Supplementary Table S3(I)). Probability to be active (Pa) and the probability to be inactive (Pi) in several biological activities were derived for vitamin K, vitamin E, ZnPT, and vitamin D by QSAR (Quantitative Structure–Activity Relationship) analysis and listed the parameters exhibiting more than 70% Pa for each adjuvant (Supplementary Table S3(II)). Moreover, Inhibition constant (K_i_) values of the vitamin K, vitamin E, ZnPT, and vitamin D were calculated against enzyme and found 0.96, 1.35, 1.60, and 10.23 µM respectively, less than the K_i_ value of 12.12 µM of acetyl-CoA (Table 1). The less K_i_ value of these compounds indicated the efficient inhibitory potential against the target enzyme compared to acetyl-CoA.

**Figure 2:**
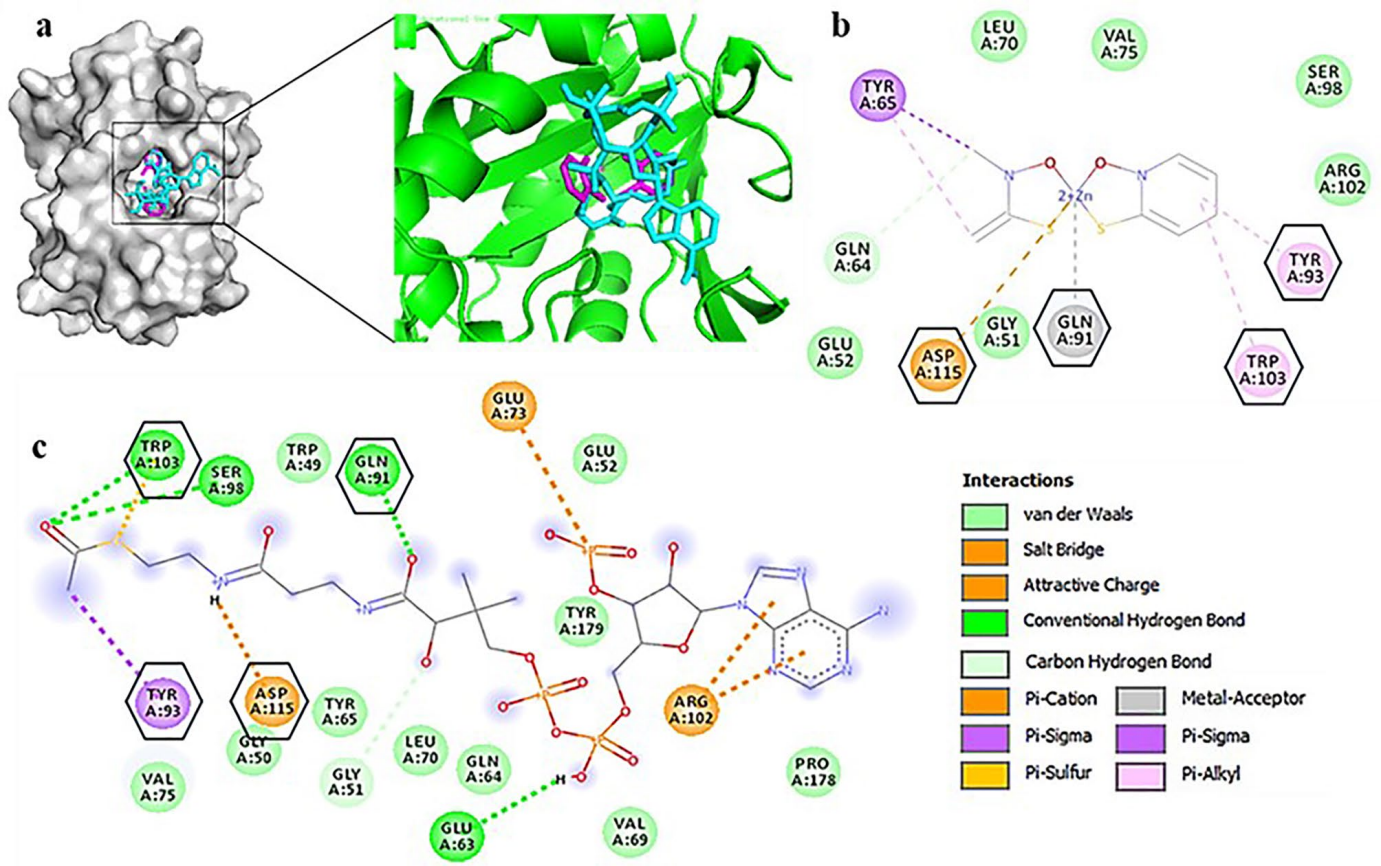
Binding mode and types of interactions between natural ligand acetyl-CoA and ZnPT with AAC(6′)-Ib of *E. coli*. Presented the summary of the docked pose of ligand acetyl-CoA (cyan) and ZnPT (magenta) in the active sites of aminoglycoside 6′-*N*-acetyltransferase (gray- whole protein, green-binding site) (**a**). Compared the binding interactions of the AAC(6′)-Ib with ZnPT and acetyl-CoA in (**b**, **c**) respectively where interacting amino acid residues are displayed and common residues between them are marked with hexagonal shapes. The types of interactions are listed at the lower-right side with. Types of interactions between enzyme and rest of the ligands are shown in Supplementary Figure S1(I). Similar types of interaction in case of other two bacteria are presented in Figure S1(II),(III).

Using PyMOL Viewer and Discovery Studio analyzer, we found that both acetyl-CoA and ZnPT bind at the same portion of the active site (Fig. 2). Resemblances of binding residues between inhibitor and substrate with enzyme theoretically confirm that the inhibitor indeed binds to the site of substrate binding resulting in the interference of substrate interactions. Some common interacting residues were Gln-91, Tyr-93, Trp-103, and Asp-115. Similarity among binding residues of vitamin D, E, and K with acetyl-CoA against target enzyme AAC (6*′*)-Ib of *E. coli* was also observed and presented in Supplementary Fig. S1(I). Binding interactions for *K. pneumoniae* and *S. sonnei* are presented in Supplementary Fig. S1(II),(III) respectively and the amino acid compositions of active site are shown in Supplementary Text S1.

### Effects of selected adjuvants in combination with antibiotics in antibiotic susceptibility test

**Figure 3:**
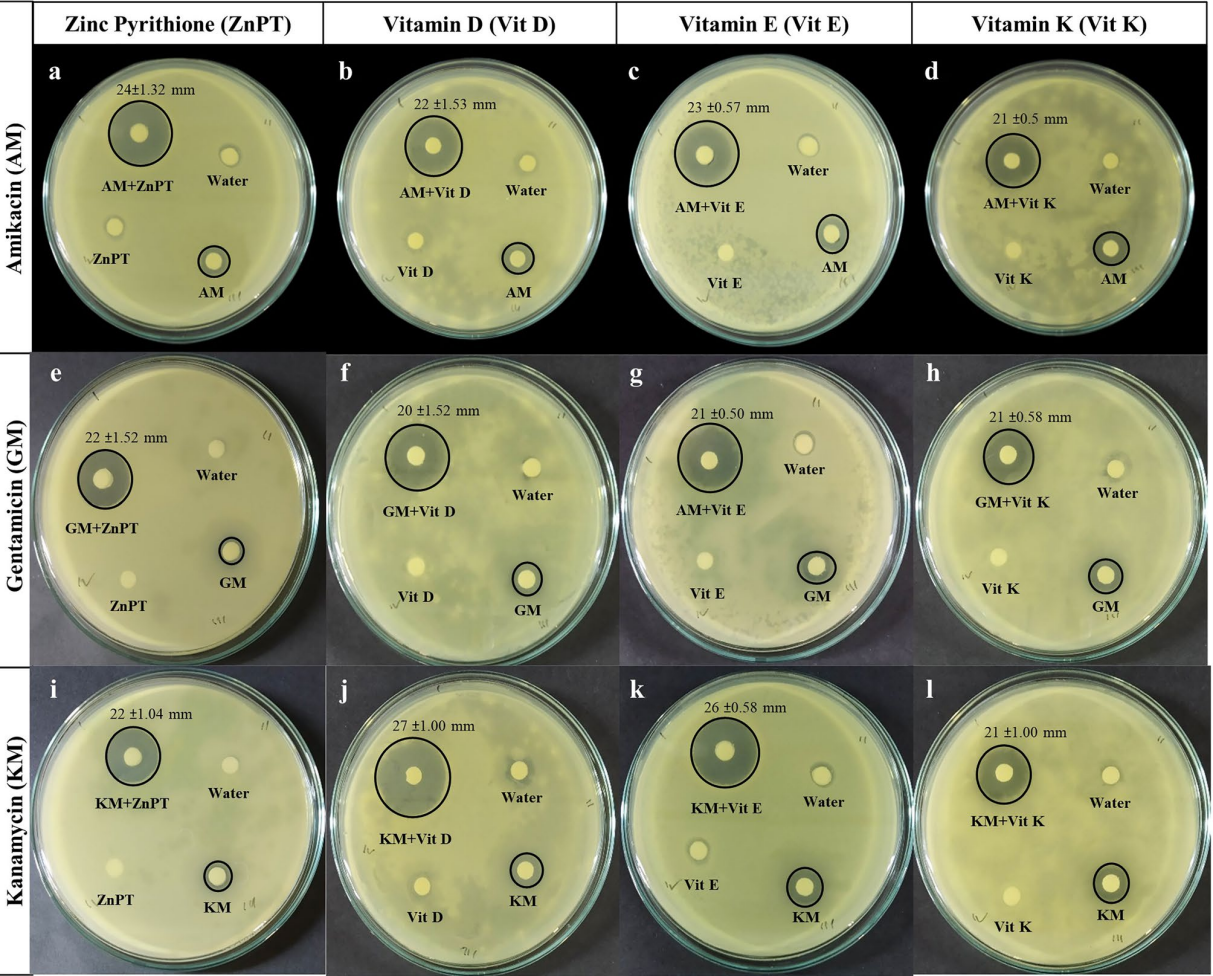
Effects of antibiotic-adjuvants on *Escherichia coli* growth in disc diffusion test. Each plate has four discs containing water, antibiotic, adjuvant and combination of antibiotic and adjuvant. In each case, the antibiotic-adjuvant combination showed a significantly large zone of inhibition compared to antibiotic and adjuvant only. Similar effects of the antibiotic-adjuvant combination on *S. sonnei* and *K. pneumoniae* are shown in Supplementary Figs. S2 and S3.

Antibiotic sensitivity test was performed to determine the resistance patterns of each bacteria against three antibiotics as well as the performances of adjuvants and antibiotics combinations. Although ZnPT alone and amikacin itself caused no significant inhibition zone, ZnPT in combination with amikacin increased the diameter of zone of inhibition to 24 ± 1.32 mm *(P* < *0.01)* in *E. coli* (Fig. 3 and Table 2), 17 ± 1.04 mm (*P* < *0.01*) in *K. pneumoniae* and 19 ± 2.00 mm (*P* < *0.01*) in *S. sonnei* compared to the control of 7 ± 0.05 mm (Supplementary Figs. S2 and S3). Similar results were also found in case of vitamins, where each of the vitamin and amikacin combinations significantly increased the inhibition zone and vitamins by themselves had no effect on bacterial growth. Gentamicin with each of three vitamin and ZnPT combinations showed an expected zone of inhibition among all three species. Same as each combination of vitamin D, vitamin E and vitamin K with kanamycin in *E. coli* seemed to be much more effective in showing an increased inhibition zone measured as 27 ± 1.00 mm (*P* < *0.01*), 26 ± 0.58 mm (*P* < *0.01*) and 21 ± 1.00 mm (*P* < *0.01*) respectively (Fig. 3, Table 2). Statistically significant inhibition zone of *K. pneumoniae* and *S. sonnei* are presented in Table 2, Supplementary Figs. S2 and S3. ZnCl_2,_ which scored lower than the acetyl-CoA in molecular docking (− 2.3 kcal/mol), was found to possess no inhibitory effects on bacterial growth since the addition of this compound did not cause any inhibition zone against bacteria (Supplementary Fig. S8).

**Table 2:** Size of inhibition zone and their corresponding statistical significance (p value) of *E. coli* (**a**), *K. pneumoniae* (**b**) and, *S. sonnei* (**c**). Zone size is expressed as mean ± standard deviation in millimeter (mm).

Antibiotic	Adjuvant	Zone size of inhibition		*p* value
		Antibiotic	Antibiotic and adjuvant	
***a) Escherichia coli***				
Amikacin	Zinc Pyrithione	7 ± 0.05	24 ± 1.32	< 0.01
	Vitamin D	7 ± 0.05	22 ± 1.53	< 0.01
	Vitamin E	7 ± 0.05	23 ± 0.57	< 0.01
	Vitamin K	7 ± 0.05	21 ± 0.50	< 0.01
Gentamicin	Zinc Pyrithione	6 ± 0.00	22 ± 1.52	< 0.01
	Vitamin D	6.4 ± 0.26	20 ± 1.52	< 0.01
	Vitamin E	7 ± 0.05	21 ± 0.50	< 0.01
	Vitamin K	7 ± 0.05	21 ± 0.58	< 0.01
Kanamycin	Zinc Pyrithione	6.2 ± 0.15	22 ± 1.04	< 0.01
	Vitamin D	6.9 ± 0.05	27 ± 1.00	< 0.01
	Vitamin E	7 ± 0.05	26 ± 0.58	< 0.01
	Vitamin K	7 ± 0.05	21 ± 1.00	< 0.01
***b) Klebsiella pneumoniae***				
Amikacin	Zinc Pyrithione	7 ± 0.05	17 ± 1.04	< 0.01
	Vitamin D	7 ± 0.05	18 ± 0.76	< 0.01
	Vitamin E	7 ± 0.05	21 ± 0.50	< 0.01
	Vitamin K	7 ± 0.05	15 ± 0.40	< 0.01
Gentamicin	Zinc Pyrithione	6.1 ± 0.15	11 ± 0.95	0.02
	Vitamin D	7 ± 0.06	18 ± 0.77	< 0.01
	Vitamin E	7 ± 0.05	19 ± 1.50	< 0.01
	Vitamin K	7 ± 0.05	20 ± 1.32	< 0.01
Kanamycin	Zinc Pyrithione	7 ± 0.05	15 ± 1.60	0.01
	Vitamin D	7 ± 0.06	18 ± 0.76	< 0.01
	Vitamin E	7 ± 0.06	20 ± 1.70	< 0.01
	Vitamin K	7 ± 0.06	24 ± 1.25	< 0.01
***c) Shigella sonnei***				
Amikacin	Zinc Pyrithione	7 ± 0.05	19 ± 2.00	< 0.01
	Vitamin D	7 ± 0.06	20 ± 0.51	< 0.01
	Vitamin E	7 ± 0.00	20 ± 1.70	< 0.01
	Vitamin K	7 ± 0.05	15 ± 0.55	< 0.01
Gentamicin	Zinc Pyrithione	7 ± 0.05	21 ± 2.08	< 0.01
	Vitamin D	7 ± 0.05	17 ± 0.64	< 0.01
	Vitamin E	7 ± 0.06	17 ± 1.55	< 0.01
	Vitamin K	6.5 ± 0.15	15 ± 1.00	< 0.01
Kanamycin	Zinc Pyrithione	6.5 ± 0.16	20 ± 0.57	< 0.01
	Vitamin D	6.2 ± 0.10	10 ± 0.58	< 0.01
	Vitamin E	6 ± 0.00	13 ± 1.00	< 0.01
	Vitamin K	6 ± 0.16	08 ± 1.15	0.05

### Bacterial growth decline in presence of antibiotic-adjuvant combinations

**Figure 4:**
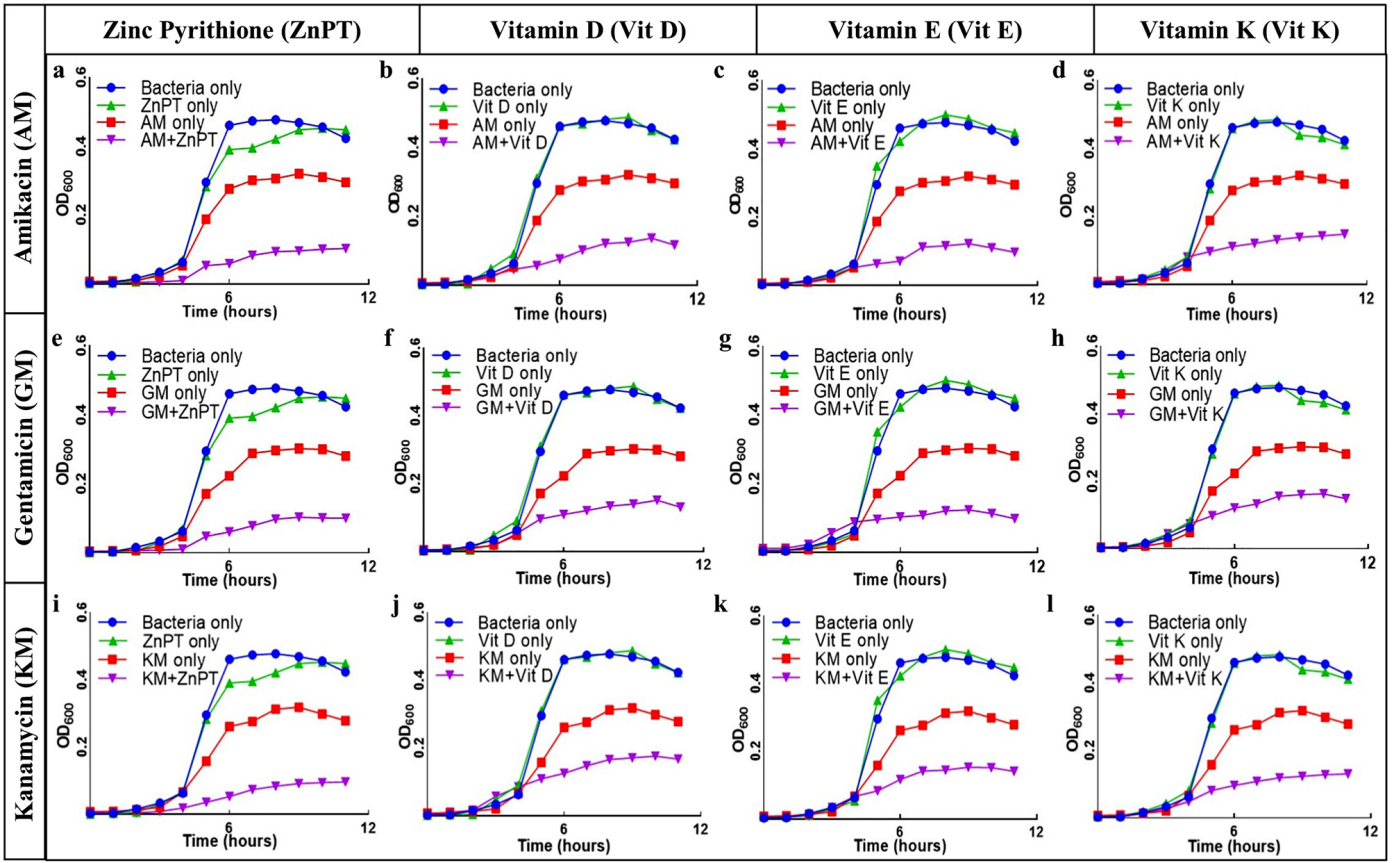
Effects of antibiotic-adjuvants on *Escherichia coli* through growth curve preparation. *E. coli* was found to be resistant to several aminoglycoside antibiotics (AM, KM, GM) but killed by their combination with ZnPT, vitamin D, vitamin E and vitamin K (**a**–**l**): *E. coli* was cultured in Mueller–Hinton broth at 37 °C, with the additions indicated in the figure and the OD_600_ was observed periodically. In each case, bacterial growth was significantly inhibited by antibiotic when adjuvant was present. Similar comparison of growth curves of *S. sonnei* and *K. pneumoniae* are shown in Supplementary Figs. S4 and S5.

According to the growth curves, ZnPT caused a slight reduction of cell number, but the effects eventually subsided at the later stage of stationary whereas the addition of amikacin did not cause any significant reduction of bacterial growth. Drastic reduction of cell number was noticed when ZnPT was added as an additional ingredient with amikacin (Fig. 4a). Growth curves produced in presence of gentamicin and kanamycin with ZnPT showed similar results to that of amikacin in all studied species (Fig. 4e,i, Supplementary Figs. S4, and S5). Combination of vitamin D with each of the antibiotics caused a marked reduction of bacterial number as the growth curve produced a nearly flat line which clearly suggests the inhibition of growth (Fig. 4b,f,j). In both *K. pneumonia* and *S. sonnei*, vitamin E had slight interference with normal growth of bacteria whereas a negligible increase of growth was observed in *E. coli.* Like other vitamins, vitamin E also caused noteworthy inhibition of bacterial growth when used with antibiotic and, greater inhibition and lag phase reduction were observed in *E. coli* compared to other vitamins (Fig. 4c,g,k). Vitamin K by itself had no impact on bacterial growth but the addition of vitamin K with kanamycin caused the highest reduction of bacterial growth (Fig. 4l) when compared to amikacin and gentamicin (Fig. 4d,h).

### Reduced activity of enzyme in presence of adjuvants

**Figure 5:**
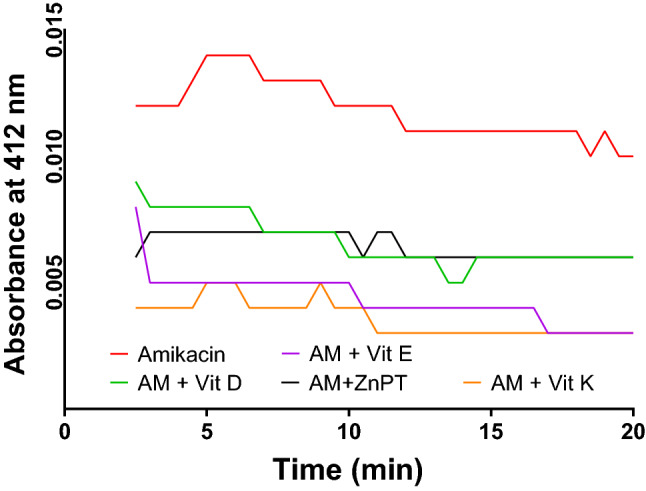
Comparative analysis of AAC(6*′*)-Ib activity in presence of amikacin (substrate) and amikacin-adjuvant combinations. In presence of amikacin as a substrate as well as positive control, the enzyme showed the highest activity (red line) through acetylation (natural ligand acetyl-CoA), whereas the combination of amikacin and adjuvants (ZnPT, vitamin D, E, and K) significantly reduced the activity of AAC(6*′*)-Ib. Similar results of enzyme activity reduction by adjuvants with Kanamycin and Gentamicin combinations are presented in Supplementary Fig. S6.

The activity of AAC(6*′*)-Ib enzyme of *E. coli* was tested in varying reaction conditions: in the presence of antibiotic and in the presence of both antibiotic and adjuvants. Antibiotics and ZnPT combination reduced the overall activity of the enzyme. All the vitamins also exhibited significant reduction in the enzyme activity. In case of amikacin, the highest level of activity reduction was observed with vitamin K, and the order of activity reduction by vitamin E, ZnPT, and vitamin D can be seen in Fig. 5.

## Discussion

Though the development of inhibitory molecules (e.g. Clavulanic acid, Colistin) against β-lactamases that confer resistance against β-lactam group antibiotics have been very intensive and successful^10,14^, but the same cannot be said for the aminoglycoside group of antibiotics. The efforts to search for inhibitors of aminoglycoside modifying enzymes have so far been insubstantial and consequently, the number of aminoglycoside adjuvants is very limited. Due to this circumstance, our study focused on identification and selection of potential enzyme inhibitors (adjuvants) through computational screening (MD method, a novel approach) and subsequent in-vitro validation to present four additional compounds (ZnPT, vitamin D, E, K) if they are capable of blocking bacterial resistance mechanism when used in combination with several aminoglycoside antibiotics.

Primarily, we conducted molecular docking of 38 different metal salts and vitamins with AAC(6*′*)-Ib enzyme of three bacterial species on the basis of few characteristics. First, they should be micronutrients and safe for human health. Second, they should have a molar mass of less than 600 Da (g/mol) so that they can cross the bacterial cell membrane^10,31^. Third, it should possess good absorption, metabolism, and excretion. Four compounds- ZnPT, vitamin D, vitamin E, and vitamin K were finally selected as they possessed higher scores of binding affinities than the natural substrate acetyl-CoA (Table 1) and had an acceptable profile both in ADMET, QSAR profiling (Supplementary Table S3(I), (II)). Higher affinity score of ZnPT and the enzyme activity assay suggests a more favorable binding of ZnPT with the modifying enzyme than acetyl-CoA strengthening the fact that ZnPT interferes with AAC(6*′*)-Ib thus preventing bacteria to confer resistance against AGs. Although in terms of drug design, energy minimization of designed ligand used to be a fact to be considered, Molecular Docking between flexible ligand, without being energetically minimized and a rigid protein receptor has been frequently used due to the advantage of a single protein receptor to recognize many dissimilar ligands^32,33^. In this study, we adopted this approach for adjuvant selection and considered as another useful strategy for efficient adjuvant selection.

Combination of ZnPT with amikacin successfully reversed the resistant profile of bacterial strains by inhibiting the growth in both solid and liquid culture media. The diameter of inhibition zone increased from resistant category to susceptible. Similar results of resistance reversal of kanamycin and gentamicin using ZnPT have also been observed. This drastic improvement of inhibition by ZnPT might have occurred due to the presence of pyrithione ionophore which may facilitate the entry of Zn^2+^ to bacterial cells^34^. Moreover, the maximum degree of growth inhibition and enzyme activity reduction in case of *E. coli* were observed by a concentration of 2 µM ZnPT (Fig. 5). Gradual increment of concentration from 2 µM reduced the inhibition zone and failed to reduce the enzyme activity as well (Supplementary Fig. S7). The possible explanation might be the formation of co-ordination complex within themselves due to the increased concentration thus preventing the exertion of inhibitory action by blocking each other’s way to the target site resulting in normal acetylation of AGs^35^.

The effects of lipid-soluble vitamins as a potentiator of resistant AGs against gram-negative bacteria were also explored in this study. The computed docking value of vitamin D, E, and K against AAC(6*′*)-Ib were higher than acetyl-CoA as of ZnPT, and none of the vitamins had any impact on bacterial growth. However, the synergistic effect of these vitamins with AGs was clearly visible and the addition of these vitamins with amikacin, gentamicin and kanamycin turned the antibiotics to sensitive for each of the studied resistant strains. Uses of multivitamins, mostly vitamin E and D along with antibiotics to treat bacterial infections have been suggested by clinicians since it boosts up the immune system by inducing the synthesis of antimicrobial peptides^36–38^. For instance, some immunomodulatory peptide such as LL-37 up-regulates the neutrophil antimicrobial responses and down-regulates pro-inflammatory cytokines and IFN-γ^39,40^. However, these studies do not deny the possibility of negative impacts of adjuvants on immune responses depending on physicochemical properties of ligand molecules. Application of vitamins as an inhibitor of aminoglycoside modifying enzymes (AMEs) explored in this study has not been reported in any previous study to the best of our knowledge. Both effects like boosting up of immune response and/or interference of bacterial resistance might be an important new finding for the medical, pharmaceutical or life sciences community for using vitamins/ZnPT (independently or cocktail form) with antibiotics after further analyses which are under research now.

Furthermore, enzymatic activity assay of AAC (6′)-Ib (Fig. 5) revealed some distinct results where the descending order of enzyme activity in presence of inhibitors-vitamin K, vitamin E, ZnPT, and vitamin D correlated with their corresponding order of MD score (ΔG = − 8.2, − 8.0, − 7.9, − 6.8 kcal/mol). The exact type of acetylation inhibition of AGs is yet to be elucidated: inhibition could occur through a competitive, non-competitive or uncompetitive mechanism. However, interaction analysis of the enzyme with both ZnPT and acetyl-CoA (Fig. 2) revealed four common interacting amino acid residues out of 12 in both ZnPT and acetyl-CoA and remaining eight amino acid residues were also found to be in the vicinity of the acetyl-CoA binding site. Similarities in the binding residues of both ZnPT and acetyl-CoA indicate the possibility of competitive inhibition which usually occurs by the accommodation of inhibitor in the substrate-binding site and their competition to bind to the enzyme’s active site.

This study gives an insight into the promising aspects of molecular docking to find potential inhibitors of resistant bacteria, with the possibility of stretching further to develop a single antimicrobial agent by targeting a conserved/common motif of the structural or resistant gene for halting the proliferation of a family or group of bacteria as a recent study exposed the bacteria to higher than adequate concentration of antibiotic to elucidate the stages of their evolution to resistance and found mutable genes as potential drug target^41^. It also opens up the opportunity for the scientific community to explore studied compounds alongside new compounds in combination with other aminoglycoside antibiotics such as streptomycin, tobramycin, etc. against the wide array of AAC(6′)-Ib enzyme-containing bacteria such as—*Acinetobacter baumannii, Pseudomonas aeruginosa, Vibrio cholera *etc. The results of this study posit ZnPT and lipid-soluble vitamins: vitamin D, E, and K as additional compounds to potentiate the antibiotic against resistant gram-negative strains by inhibiting AAC(6*′*)-Ib enzyme. We are optimistic that this new approached article will stimulate interest in using those combined in silico and in vitro methods to develop agents in the war against antibiotic resistance.

## Methods

### Ligand selection, preparation, ADMET, and QSAR profiling

Thirty-eight different chemical compounds were chosen as the ligand for molecular docking. Ligands were primarily screened based on few important criteria: have to be nontoxic, molecular weight of less than 600 Da (g/mol) and good absorption, metabolism, and excretion status. Therefore, ADMET (absorption, distribution, metabolism and toxicity) profiling (https://lmmd.ecust.edu.cn/admetsar2/) and QSAR (Quantitative Structure–Activity Relationship) analysis (https://www.pharmaexpert.ru/passonline/) were carried out for this purpose. All chemical structures were retrieved from the NCBI PubChem database (https://pubchem.ncbi.nlm.nih.gov/). All of the downstream file conversion necessary for molecular docking was performed by the open-access chemical toolbox OpenBabel v 3.0.0^42^.

### Protein model preparation and structural validation

The protein sequences of AAC(6′)-Ib enzyme of three bacterial species including *E. coli, K. pneumoniae,* and *S. sonnei* were retrieved from the NCBI database. Several physical and chemical properties of the respective enzyme were determined by the bioinformatic tool ProtParam^43^ (https://web.expasy.org/protparam/) and NetPhos 3.1^44^ server (https://www.cbs.dtu.dk/services/NetPhos/). The crystal structure of AAC(6′)-Ib enzyme of *E. coli* was retrieved from RCSB Protein Data Bank (PDB) database (https://www.rcsb.org/) using the PDB ID 1V0C. Three-dimensional structures of AAC(6′)-Ib enzyme of *K. pneumoniae* and *S. sonnei* were constructed by Homology Modeling. Structure of each bacterial protein was modeled by SWISS-MODEL^45^ (https://swissmodel.expasy.org/) using their respective protein sequence. Validation of homology modeling was carried out by Ramachandran plot using PROCHECK (https://servicesn.mbi.ucla.edu/PROCHECK/) (Supplementary Table S1). We included sequence similarities, Global Model Quality Estimation (GMQE), QMEAN Z-scores, percentages of most favored, allowed and forbidden regions for determining the quality of the protein models.

### Determination of active site residues

Active site residues of each protein structure were identified to perform site-specific molecular docking. Amino acid residues of the active site were determined by the COACH^46^ server (https://zhanglab.ccmb.med.umich.edu/COACH/), an open-source meta-server for protein–ligand binding site prediction. Specific amino acid residues involved in protein–ligand interaction of *E. coli* AAC(6′)-Ib predicted by the COACH server were also confirmed by analyzing the binding residues of the natural substrate and target protein complex available in the PDB structure.

### Grid box setting, molecular docking, and inhibition constant (K_i_) calculation

Molecular docking was executed to determine the binding affinity (ΔG in kcal/mol) between ligand and the target protein. An open-source molecular docking program AutoDock Vina v 1.1.6^30^ was employed to calculate the protein–ligand binding interactions. Grid box setting is a crucial step in molecular docking which instructs the docking program to search for any available ligand–protein interactions in a user-defined search space of target protein. In this regard, grid box coordinates of each of the three AAC(6′)-Ib (Table 3) were defined in such a way, likewise in blind docking, to accommodate all of the active site residues of target receptors within the search box for the candidate ligand. First, the box dimensions in *x*, *y* and *z* were increased by 10 Å. Additionally, one of the two directions in each dimension was randomly chosen and further increased by 5 Å. Finally, the box size of 80 Å, 100 Å, 96 Å in the x, y, and z dimension respectively was set for the active site of the protein of *E. coli*. Similar setting was followed for the protein of two other species. Followed by the grid box setting, spacing was maintained at 1.00 Å and exhaustiveness value was set at default 8 in throughout all docking run. All of the essential parameters were applied on all ligands and receptors by AutoDock Tools v 4.2^47^. Non-rotatable bonds present in the any of the ligands were treated as rotatable to allow flexibility of the ligand. Any available water molecules and hetero-atoms in the target protein structures were removed to prevent any redundant interference with the ligand–protein interaction. Since the PDB structures do not generally contain any hydrogen atoms and charge, polar hydrogens were added in the protein structure to account for any possible hydrogen bonding between ligand and receptor and to emulate in-vivo environment. All of the ligands were ranked based on their corresponding affinity value with each of the target enzymes. Inhibition constant (K_i_) of the docked compounds have been calculated from the affinity score by applying the following formula:

**Table 3:** Grid box parameters of the target enzymes defined for molecular docking.

AAC(6′)-Ib of bacteria	Center of the grid box (points in X, Y and, Z axis)	Size of the grid box (points in X, Y and, Z axis)
*E. coli*	− 6.728, 16.206, − 18.199	80, 100, 96
*K. pneumoniae*	0.162, − 20.037, − 17.809	100, 94, 86
*S. sonnei*	0.167, − 20.032, − 17.802	100, 96, 98

$$Inhibition\;constant\;\left({\mathrm{K}}_{i}\right)\;= {exp ( }_{R*T}^{\Delta G})$$

where ΔG denotes binding affinity in Cal/mol, R denotes gas constant (1.986 Cal/mol-K), and T represents temperature in Kelvin (298 K). Generated output files of the docking program were visualized by PyMol^48^ and intermolecular interactions between ligand and receptor protein were analyzed by BIOVIA Discovery Studio Visualizer v 19.1.0^49^.

### Antibiotic susceptibility test and statistical analysis of zone of inhibition

Antimicrobial susceptibility test was performed to assess the effects of several ligand compounds as antibiotic adjuvant on bacterial growth. Bacterial growth was monitored by two methods: broth culture and disc diffusion test. Three gram-negative bacterial strains namely *E. coli, K. pneumoniae,* and *S. sonnei* were selected for the study. Each of the bacterial strains was cultured in four different culture conditions. They were: without any antibiotic and adjuvant (only wild bacteria), with only antibiotic, with only adjuvant and, with antibiotic and adjuvant combination. Three different aminoglycoside antibiotics including amikacin (30 µg), gentamicin (30 µg) and, kanamycin (30 µg) were used in this study. Antibiotic disc concentration was selected according to the common clinical laboratory practice and The Clinical & Laboratory Standards Institute (CLSI) guidelines. Four different compounds were selected as adjuvants based on their docking score higher than the enzyme’s natural substrate acetyl-CoA. They were: zinc pyrithione (2 µM), vitamin D (125 µg/ml), vitamin E (200 IU) and, vitamin K (100 µg/ml). Bacteria were cultured in LB broth medium at 37 °C with varying culture conditions mentioned above and the growth was observed by monitoring the change of optical density (OD_600_) of culture media measured spectrophotometrically. Similar tests were also carried out in solid bacterial media. Bacteria were cultured in Mueller Hinton (MH) agar media in presence of only antibiotic, only adjuvant and, antibiotic adjuvant combination. Sterile blank filter discs with a diameter of 6 mm were soaked with the aforementioned chemical compounds and disc containing water was used as negative control. Zone of inhibition caused by the discs was measured in millimeter (mm). Significance of the differences between the zone size of antibiotic and antibiotic-adjuvant combination was calculated through paired sample t-test analysis using IBM SPSS Statistics V.25. Additionally, from the ligands with the affinity score lower than the acetyl-CoA, ZnCl_2_ (0.1 M) was used to check its ability to retain antibiotic activity.

### Extraction of enzyme and enzyme activity assay

To induce the synthesis of AAC(6′)-Ib enzyme in bacteria, *E. coli* was allowed to grow in appropriate growth media at 37 °C in the presence of antibiotic till the late logarithm phase. AAC (6′)-Ib enzyme was extracted and purified in specific buffer [20 mM Tris–Cl (pH 7.5), 10 mM MgCl_2_, 30 mM NH_4_Cl, and 2-mercaptoethanol]. Usually, in the presence of antibiotic, bacterial AAC(6′)-Ib transfers acetyl group from its own acetyl-CoA generated by cellular metabolism to the substrate antibiotic. This acetylation reaction generates a free sulfhydryl (–SH) group as an end product. In our experiment, a free sulfhydryl group corresponds to the acetylation of antibiotic which was determined by the addition of Ellman’s reagent [5,5′-dithiobis 2-nitrobenzoic acid (DTNB)]. Addition of DTNB to the reaction mixture containing free -SH group increases the absorption at 412 nm. The increase of absorption was monitored spectrophotometrically to measure the enzymatic activity of AAC(6′)-Ib. Each reaction mixture was composed of appropriate buffer solution (10 mM Tris–Cl, MgCl_2_, and EDTA), 500 µM DTNB (Ellman’s Reagent)^34,50^, 150 µM acetyl-CoA, and antibiotic solution (amikacin or gentamicin or kanamycin). Acetyl-CoA was provided as acetyl group donor to the AAC(6′)-Ib enzyme since the bacteria which usually source it from its regular metabolism was absent in the reaction mixture. Reactions were initiated by the addition of the extracted enzyme and the absorbance was recorded for 20 min after a brief incubation period of 2 min at room temperature. To examine the inhibitory effects of inhibitors, AAC(6′)-Ib activity was measured in a fresh reaction mixture containing the aforementioned ingredients along with one of the four inhibitors (ZnPT or Vitamin D or Vitamin E or Vitamin K).

### Source of the chemicals

All the chemicals used in this study were of analytical grade and experiments were carried out at biosafety level I facility. The source includes: Luria Bertani Broth (M1245, HiMedia Laboratories, India), Luria Bertani Agar (M1151, HiMedia Laboratories, India), Acetyl-CoA (10101893001, Roche Diagnostics GmbH, Germany), Elmann’s Reagent (32363, Sisco Research Laboratories Pvt. Ltd., India), Antibiotic Discs (Thermo Scientific Pvt. Ltd.).

## Supplementary information

Supplementary Information.
