# Supplementary material for: Retention of antibiotic activity against resistant bacteria harbouring aminoglycoside-N-acetyltransferase enzyme by adjuvants: a combination of in-silico and in-vitro study
Source: Sci Rep. 2020 Nov 9;10:19381. doi: 10.1038/s41598-020-76355-0 (PMC7653040; doi:10.1038/s41598-020-76355-0)
Supplement: Supplementary file 1 — Supplementary Information. [file 41598_2020_76355_MOESM1_ESM.pdf]

Title: Retention of antibiotic activity against resistant bacteria harbouring aminoglycoside-N-acetyltransferase enzyme by adjuvants: A combination of *in-silico* and *in-vitro* study

Authors' name:

Shamim Ahmed[1*], Sabrina Amita Sony[1], Md. Belal Chowdhury[1], Md. Mahib Ullah[1], Shatabdi Paul[1], Tanvir Hossain[1]

Affiliation:

[1]*School of Life Sciences, Department of Biochemistry and Molecular Biology, Shahjalal University of Science and Technology, Sylhet 3114, Bangladesh.*

[*]Corresponding author:

Shamim Ahmed, PhD

Associate Professor

Department of Biochemistry and Molecular Biology,

Shahjalal University of Science and Technology, Sylhet 3114, Bangladesh

E-mail: shamim1174-bmb@sust.edu, shamim1174@gmail.com

Telephone: +880 1776196006

Supplementary data

Table S1: Homology modeling-derived protein structure validation by Ramachandran plot: a) *K. pneumoniae* and b) *S. sonnei*.

a) Klebsiella pneumoniae	
**Parameter**	**Obtained Value**
Sequence Identity	97.85%
GMQE	0.92
QMEAN- Z Score	1.17
Most Favored Regions	96%
Allowed Regions	4.0%
Disallowed Regions	0.0%

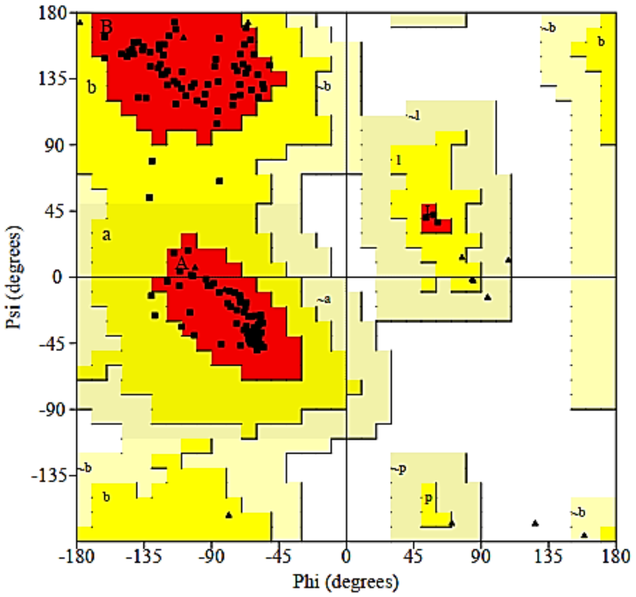

b) Shigella Sonnei	
**Parameter**	**Obtained Value**
Sequence Identity	98.91%
GMQE	0.99
QMEAN- Z Score	1.30
Most Favored Regions	96.7%
Allowed Regions	3.3%
Disallowed Regions	0.0%

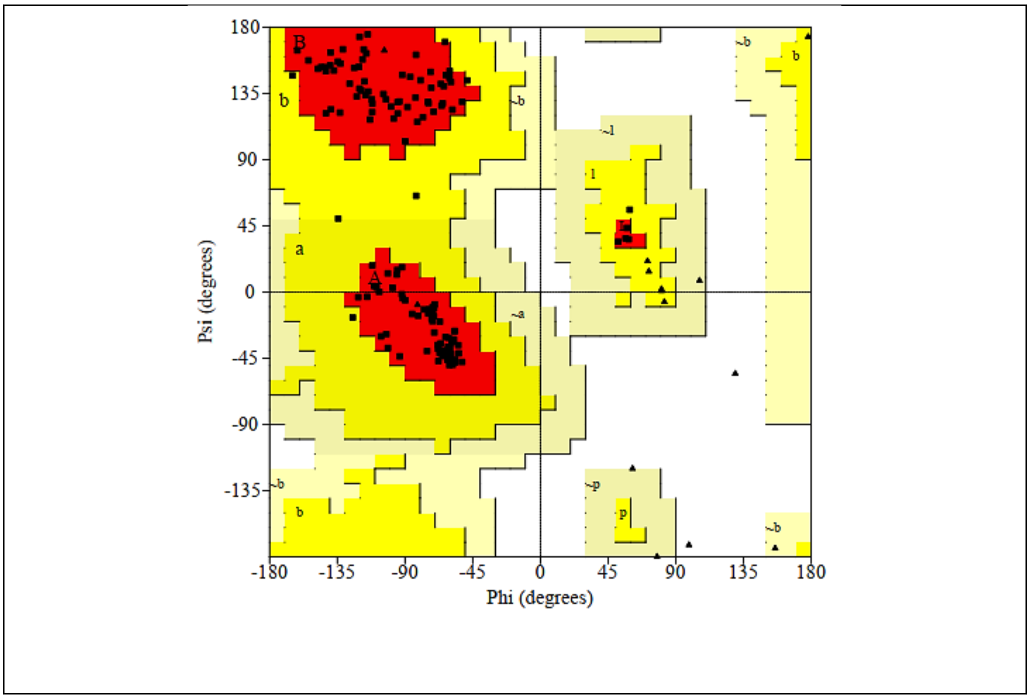

Psi (degrees)
Phi (degrees)
180
135
90
45
0
-45
-90
-135
-180
-135
-90
-45
0
45
90
135
180

4

Table S2: Computed binding affinities between enzyme and ligands. Each run of the docking generated several binding modes with corresponding docking score from which the best scored one was considered.

Bacterial Species	Mode	Affinity, ΔG (kcal/mol)				
		**Acetyl-CoA**	**Zinc Pyrithione**	**Vitamin D**	**Vitamin E**	**Vitamin K**
Escherichia coli	1	-6.7	-7.9	-6.8	-8.0	-8.2
	2	-6.4	-7.7	-6.2	-7.8	-6.7
	3	-5.7	-7.5	-4.8	-6.9	-6.4
	4	-5.3	-7.0	-4.7	-6.8	-5.7
	5	-5.0	-6.9	-4.7	-6.7	-5.6
	6	-5.0	-6.8	-4.7	-6.4	-5.5
	7	-5.0	-6.5	-4.6	-6.1	-5.4
	8	-5.0	-6.5	-4.6	-6.0	-5.4
	9	-4.9	-6.4	-4.6	-5.9	-5.3
Klebsiella pneumoniae	1	-6.9	-7.1	-7.1	-8.1	-7.1
	2	-6.8	-6.8	-6.1	-7.9	-7.1
	3	-6.7	-6.3	-6.0	-7.8	-7.0
	4	-6.6	-5.9	-5.9	-7.7	-7.0
	5	-6.5	-5.9	-5.9	-7.6	-6.9
	6	-6.5	-5.7	-5.9	-7.6	-6.8
	7	-6.2	-5.7	-5.8	-7.5	-6.7
	8	-6.2	-5.6	-5.7	-7.4	-6.6
	9	-6.1	-5.6	-5.6	-7.4	-6.5
Shigella sonnei	1	-6.0	-6.9	-8.8	-8.3	-7.3
	2	-5.3	-6.8	-8.7	-8.2	-7.1
	3	-5.3	-6.8	-8.2	-7.9	-7.0
	4	-5.3	-6.6	-8.2	-7.8	-7.0
	5	-5.1	-6.5	-7.7	-7.7	-7.0
	6	-5.0	-6.5	-7.3	-7.5	-6.7
	7	-5.0	-6.4	-6.4	-7.5	-6.7
	8	-4.9	-6.4	-6.2	-7.5	-6.6
	9	-4.7	-6.4	-6.2	-7.3	-6.6

Table S3(I): Enlisted Absorption, Distribution, Metabolism and Toxicity (ADMET) profile parameters of Zinc Pyrithione, Vitamin D, Vitamin E and Vitamin K.

Models	Zinc Pyrithione	Vitamin D	Vitamin E	Vitamin K
**A (Absorption)**				
Human Intestinal Absorption (HIA)	HIA+	HIA+	HIA+	HIA+
Blood Brain Barrier(BBB)	BBB+	BBB+	BBB+	BBB+
Human Oral Bioavailability (HOB)	HOB+	HOB+	HOB+	HOB+
Caco-2 Permeability	Caco2+	Caco2+	Caco2+	Caco2+
P-glycoprotein Substrate	Non-Substrate	Non-Substrate	Non-Substrate	Non-Substrate
P-glycoprotein Inhibitor	Non-Inhibitor	Non-Inhibitor	Non-Inhibitor	Inhibitor
**D (Distribution)**				
Subcellular Localization	Mitochondria	Lysosome	Mitochondria	Mitochondria
Plasma Protein Binding (PPB)	100%	100%	100%	100%
**M (Metabolism)**				
CYP450 3A4 Substrate	Non-Substrate	Substrate	Substrate	Substrate
CYP450 2C9 Substrate	Non-Substrate	Non-Substrate	Non-Substrate	Non-Substrate
CYP450 2D6 Substrate	Non-Substrate	Non-Substrate	Substrate	Non-Substrate
CYP450 3A4 Inhibitor	Non-Inhibitor	Non-Inhibitor	Non-Inhibitor	Non-Inhibitor
CYP450 2C9 Inhibitor	Non-Inhibitor	Non-Inhibitor	Non-Inhibitor	Inhibitor
CYP450 2C19 Inhibitor	Inhibitor	Non-Inhibitor	Non-Inhibitor	Inhibitor
CYP450 2D6 Inhibitor	Non-Inhibitor	Non-Inhibitor	Non-Inhibitor	Non-Inhibitor
CYP450 1A2 Inhibitor	Inhibitor	Non-Inhibitor	Non-Inhibitor	Inhibitor
**T (Toxicity)**				
Hepatotoxicity	Nontoxic	Nontoxic	Nontoxic	Nontoxic
Ames mutagenesis	Non-Ames toxic	Non-Amestoxic	Non-Ames toxic	Non-Ames toxic
Carcinogenesis	Non-Carcinogens	Non-Carcinogens	Non-Carcinogens	Non-Carcinogens

Table S3(II): Quantitative Structure-Activity Relationship (QSAR) profile of ZnPT, Vitamin D, E and K. Pa and Pi indicate the probability to be active and probability to be inactive repectively in biological activities.

Zinc Pyrithione		
**Activity**	**Pa**	**Pi**
Coagulant	0.882	0.001
Plastoquinol-plastocyanin reductase inhibitor	0.881	0.001
Hemostatic	0.877	0.002
Vitamin K-like	0.860	0.001
Ubiquinol-cytochrome-c reductase inhibitor	0.842	0.019
Testosterone 17beta-dehydrogenase (NADP+) inhibitor	0.790	0.028
Lipid peroxidase inhibitor	0.763	0.004
Anti-secretoric	0.760	0.005
CDP-glycerol glycerol-phosphotransferase inhibitor	0.779	0.035
CYP2J substrate	0.762	0.031
Muscular dystrophy treatment	0.709	0.003
VitaminK-epoxide reductase (warfarin-insensitive) inhibitor	0.702	0.004
TP53 expression enhancer	0.720	0.022
Apoptosis agonist	0.710	0.014
Mucomembranous protector	0.737	0.041
**Vitamin D**		
**Activity**	**Pa**	**Pi**
Antieczematic	0.949	0.003
Dermatologic	0.936	0.003
Antiosteoporotic	0.934	0.003
Proliferative diseases treatment	0.932	0.002
Vitamin	0.929	0.000
Bone diseases treatment	0.929	0.003
Antipsoriatic	0.917	0.002
Respiratory analeptic	0.906	0.004
Adenomatous polyposis treatment	0.894	0.001
CYP3A4 inducer	0.886	0.003
Analeptic	0.786	0.005
Choleretic	0.740	0.003
Acylcarnitine hydrolase inhibitor	0.885	0.005
Alkenylglycerophosphocholine hydrolase inhibitor	0.887	0.006
Alkylacetylglycerophosphatase inhibitor	0.876	0.004
CYP3A4 substrate	0.878	0.007
CYP3A inducer	0.872	0.003
CYP27 substrate	0.863	0.000
CYP27A substrate	0.847	0.000
Chemopreventive	0.849	0.003
CYP3A substrate	0.850	0.009

Antipruritic	0.823	0.003
Hyperparathyroidism treatment	0.813	0.000
CYP27A1 inhibitor	0.809	0.000
Cholestanetriol 26-monooxygenase inhibitor	0.797	0.004
DELTA14-sterol reductase inhibitor	0.786	0.002
Testosterone 17beta-dehydrogenase (NADP+) inhibitor	0.784	0.029
Apoptosis agonist	0.759	0.010
CYP27A1 substrate	0.735	0.001
Antipruritic. allergic	0.727	0.002
Antiinflammatory	0.702	0.015
**Vitamin E**		
**Activity**	**Pa**	**Pi**
Lipid peroxidase inhibitor	0.970	0.002
Antioxidant	0.967	0.002
CYP2C12 substrate	0.955	0.004
Acute neurologic disorders treatment	0.935	0.004
Anti-hypercholesterolemic	0.932	0.003
Anti-ischemic. cerebral	0.932	0.005
Reductant	0.924	0.003
AR expression inhibitor	0.851	0.002
Hypolipemic	0.846	0.005
Male reproductive disfunction treatment	0.842	0.003
Nucleotide metabolism regulator	0.829	0.005
Prion diseases treatment	0.816	0.002
Anti-inflammatory	0.814	0.006
Cholesterol antagonist	0.799	0.005
Anticataract	0.792	0.002
CYP2C19 substrate	0.793	0.004
CYP2E substrate	0.776	0.005
CYP2E1 substrate	0.775	0.005
CYP2C substrate	0.780	0.011
CYP2C8 inhibitor	0.760	0.003
CYP2C8 substrate	0.760	0.007
Ubiquinol-cytochrome-c reductase inhibitor	0.776	0.041
Apoptosis agonist	0.736	0.012
Vitamin	0.713	0.001
Glycogen synthase stimulant	0.704	0.002
Antianginal	0.709	0.008
Testosterone 17beta-dehydrogenase (NADP+) inhibitor	0.738	0.043
TP53 expression enhancer	0.712	0.024
**Vitamin K**		
**Activity**	**Pa**	**Pi**
Electron-transferring-flavoprotein dehydrogenase inhibitor	0.835	0.003
Chenodeoxycholoylta urine hydrolase inhibitor	0.824	0.003
Pro-opiomelanocortin converting enzyme inhibitor	0.789	0.016
Polyneuridine-aldehyde esterase inhibitor	0.771	0.003

Glycosylphosphatidylinositol phospholipase D inhibitor	0.779	0.017
Metalloproteinase-9 inhibitor	0.753	0.003
Dehydro-L-gulonate decarboxylase inhibitor	0.753	0.015
Pseudolysin inhibitor	0.746	0.012
Nicotinic alpha6beta3beta4alpha5 receptor antagonist	0.750	0.021
IgA-specific metallo endopeptidase inhibitor	0.730	0.006
Alkane 1-monooxygenase inhibitor	0.725	0.013
Membrane permeability inhibitor	0.734	0.026
Carboxypeptidase Taq inhibitor	0.722	0.016
Phthalate 4.5-dioxygenase inhibitor	0.707	0.014
Glutathione thiolesterase inhibitor	0.705	0.018
Aspulvinonedimethylallyltransferase inhibitor	0.729	0.054
Anti-schemic. cerebral	0.990	0.002
Polarisation stimulant	0.759	0.003
Myocardial infarction treatment	0.830	0.003
Anti-seborrheic	0.835	0.013

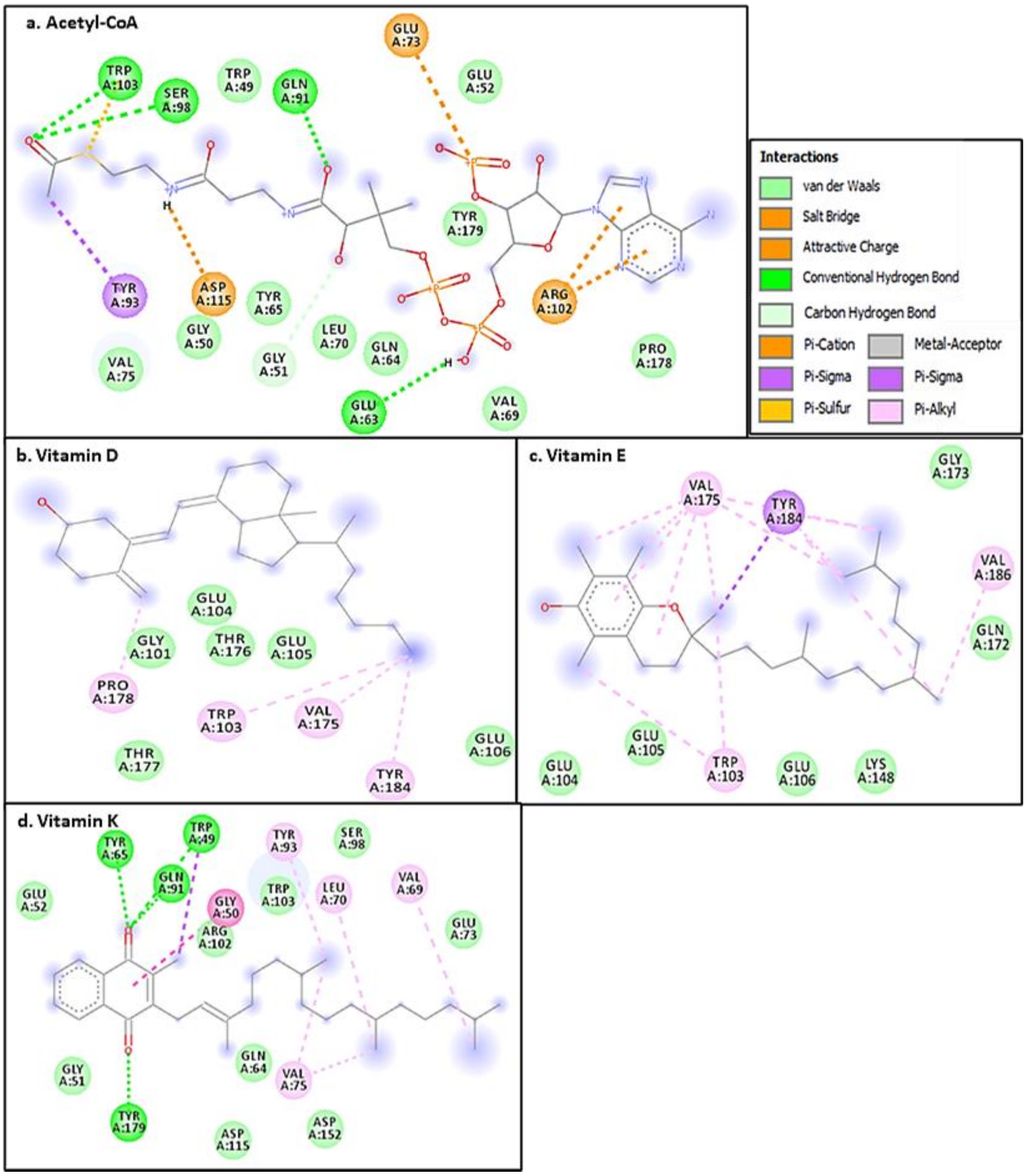

Figure S1(I): Interaction of AAC(6´)-Ib enzyme of *E. coli* with Acetyl CoA (**a**), Vitamin D (**b**), Vitamin E (**c**), and Vitamin K (**d**).

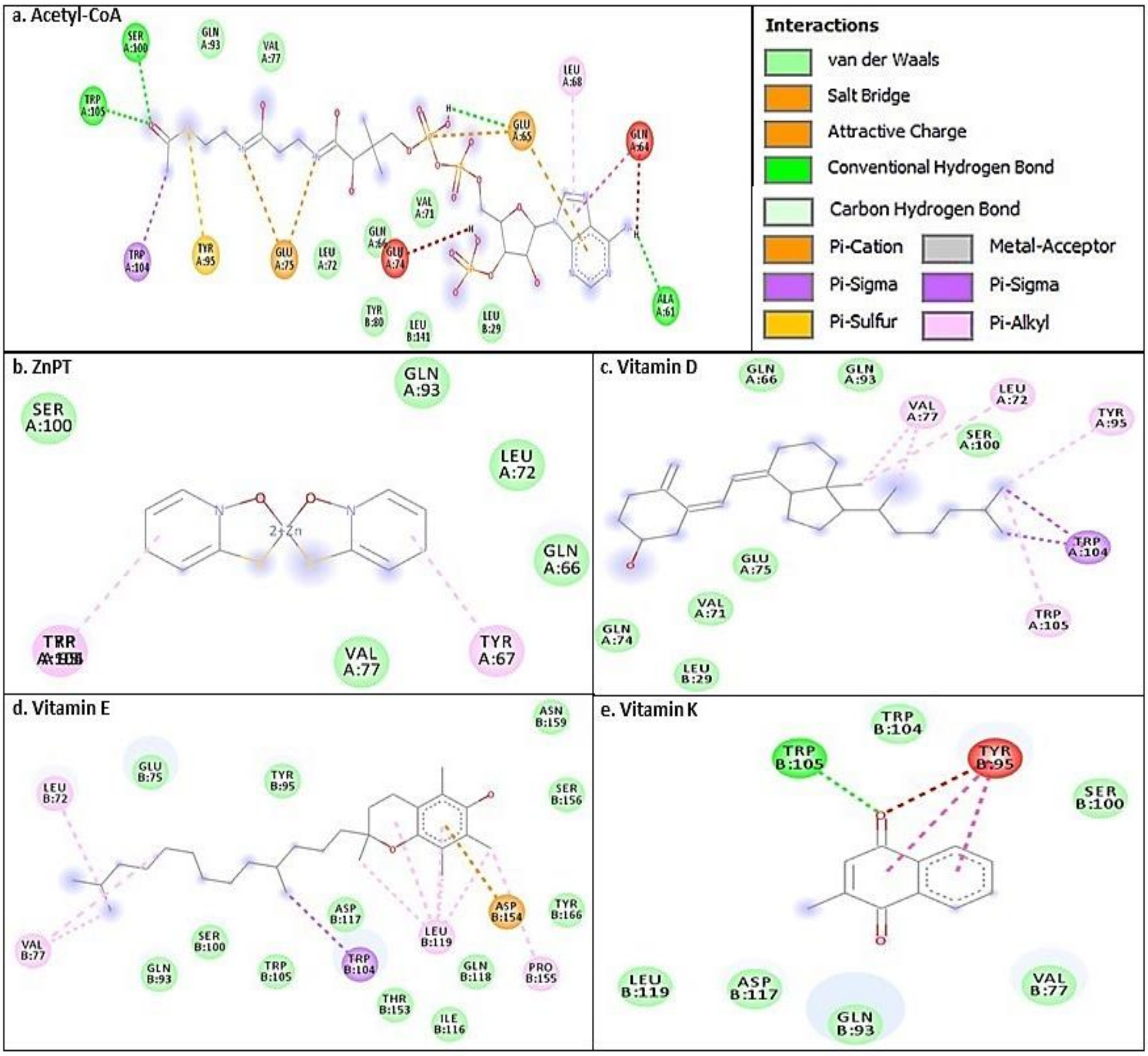

Figure S1(II): Interaction of AAC(6´)-Ib enzyme of *K. pneumoniae* with Acetyl CoA **(a)**, Zinc Pyrithione **(b)** Vitamin D **(c)**, Vitamin E **(d)**, and Vitamin K **(e).**

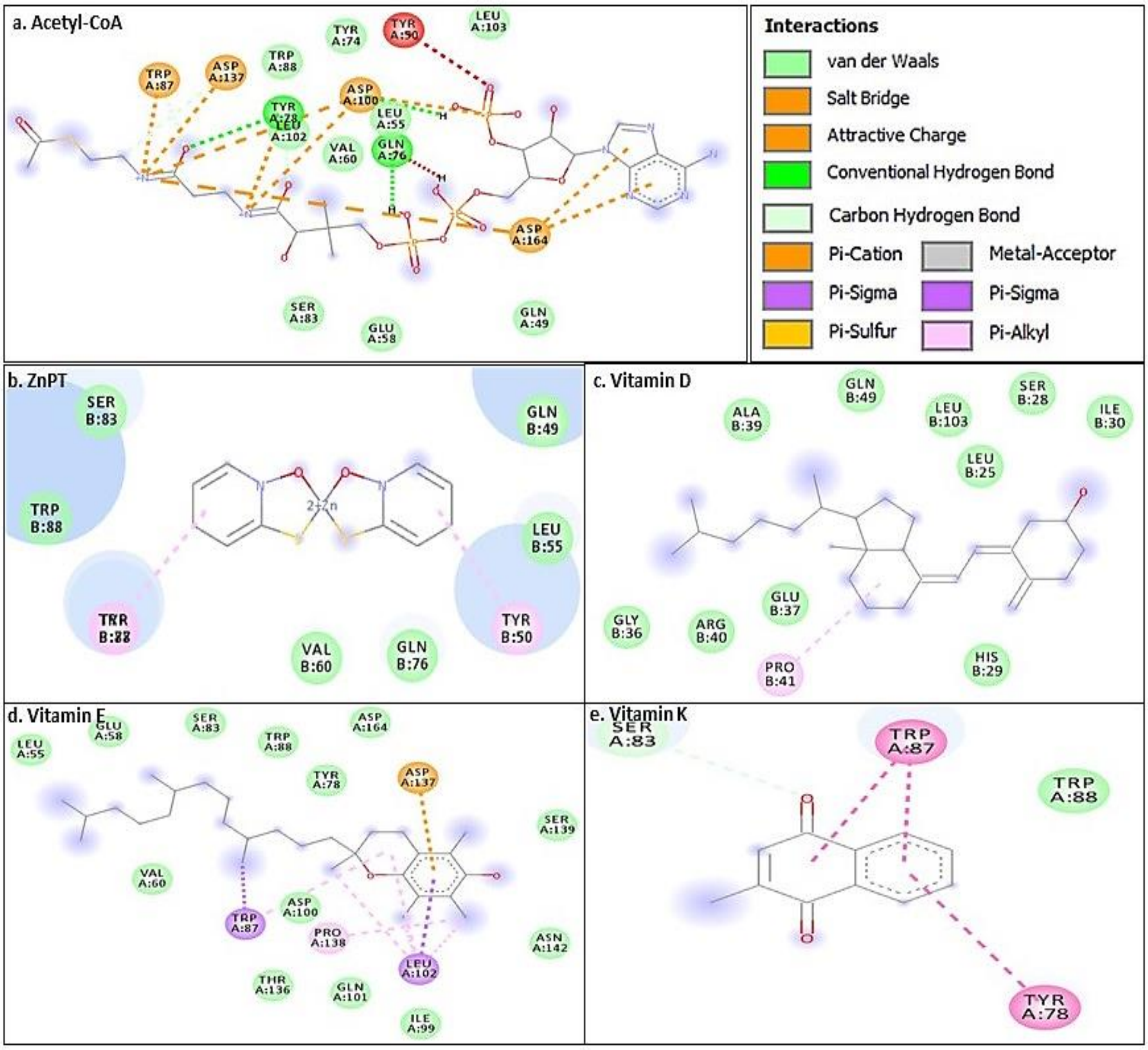

Figure S1(III): Interaction of AAC(6´)-Ib enzyme of *S. sonnei* with Acetyl CoA **(a)**, Zinc Pyrithione **(b)** Vitamin D **(c)**, Vitamin E **(d)** and, Vitamin K **(e)**.

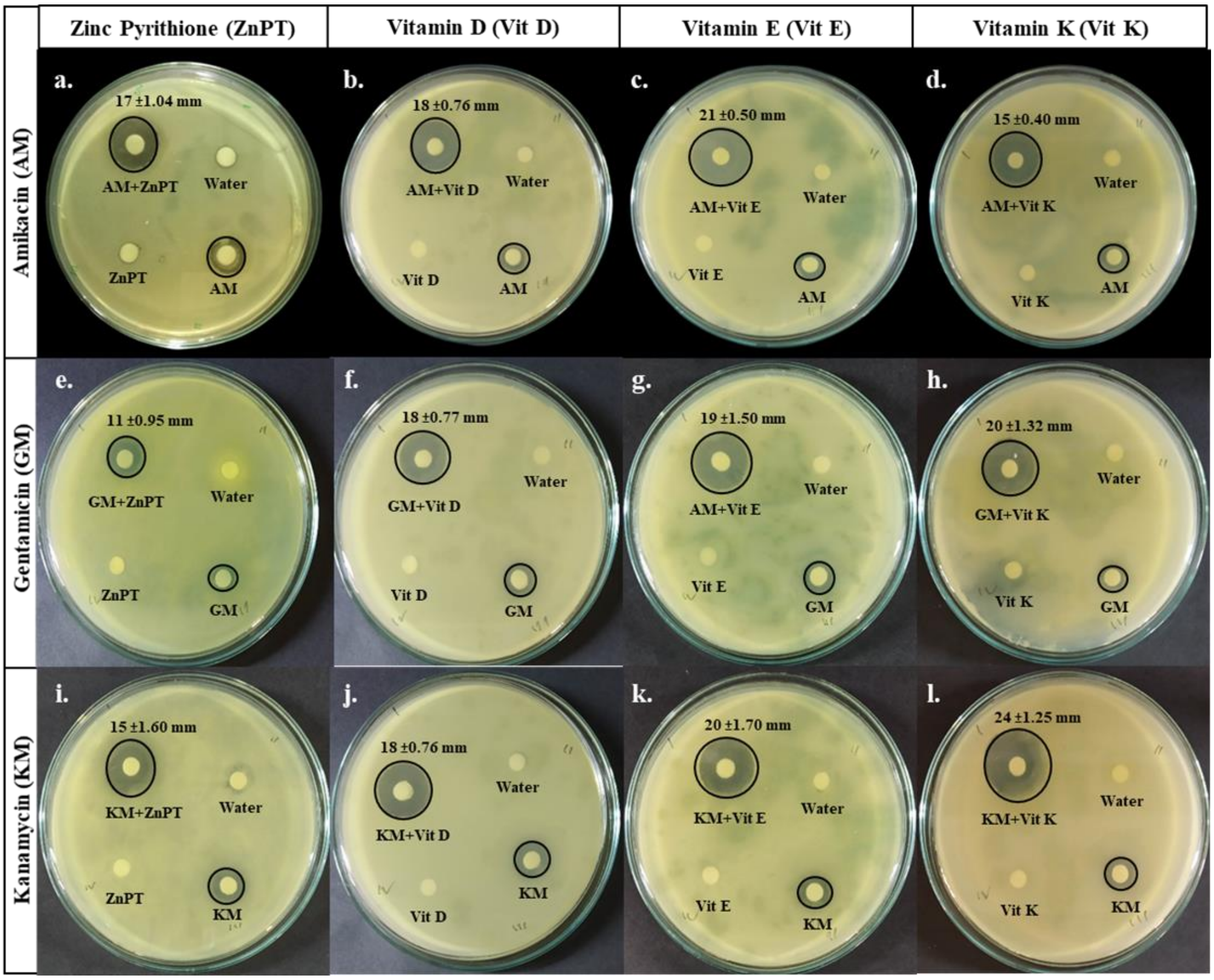

Figure S2: Effects of antibiotic-adjuvants on *Klebsiella pneumoniae* growth by disc diffusion method. Each plate has four discs containing water, antibiotic, adjuvant and a combination of antibiotic and adjuvant. In each case, the antibiotic-adjuvant combination showed a significantly larger zone of inhibition compared to antibiotic and adjuvant only.

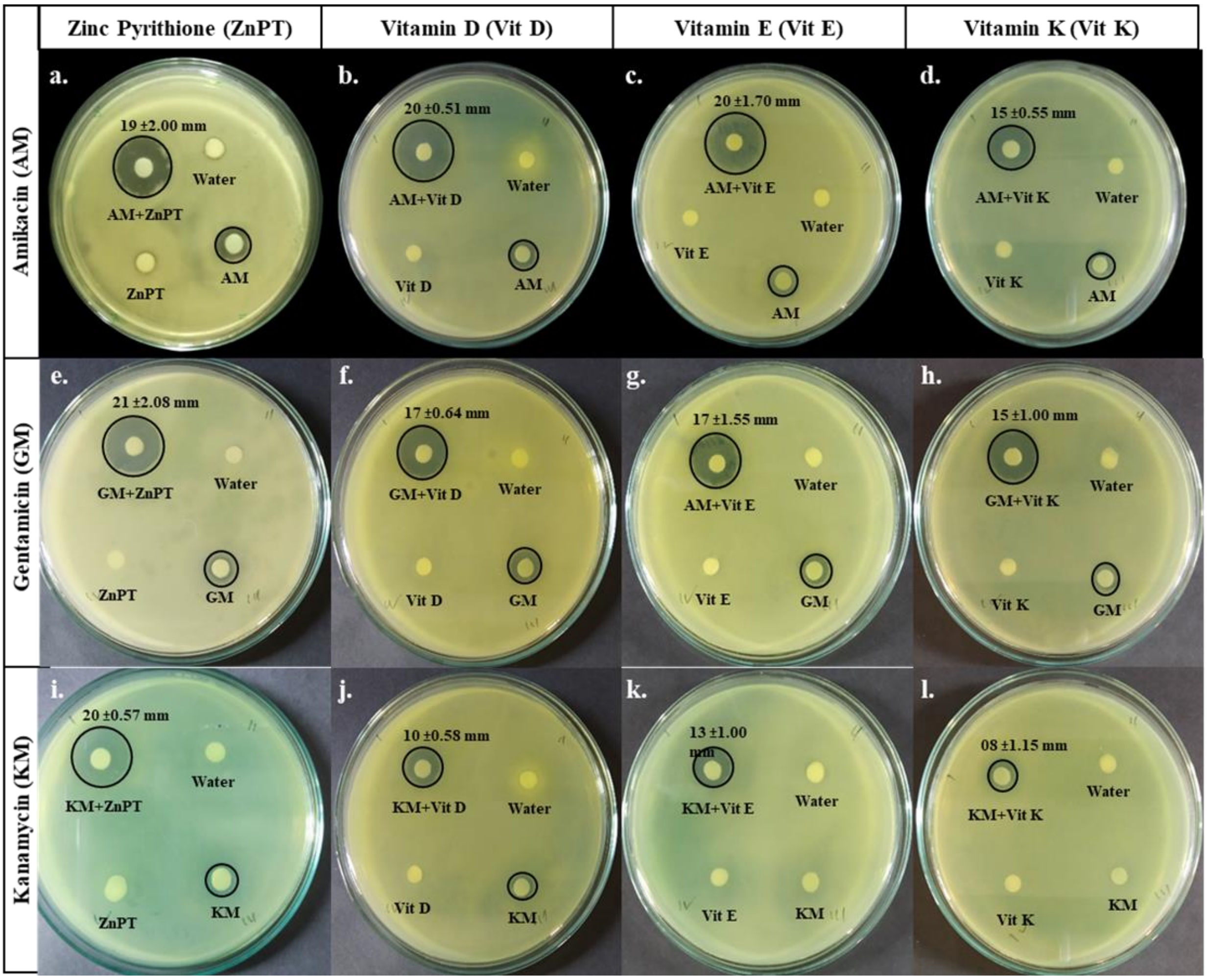

Figure S3: Effects of antibiotic-adjuvants on *Shigella sonnei* growth by disc diffusion method. Each plate has four discs containing water, antibiotic, adjuvant and a combination of antibiotic and adjuvant. In each case, the antibiotic-adjuvant combination showed a significantly larger zone of inhibition compared to antibiotic and adjuvant only.

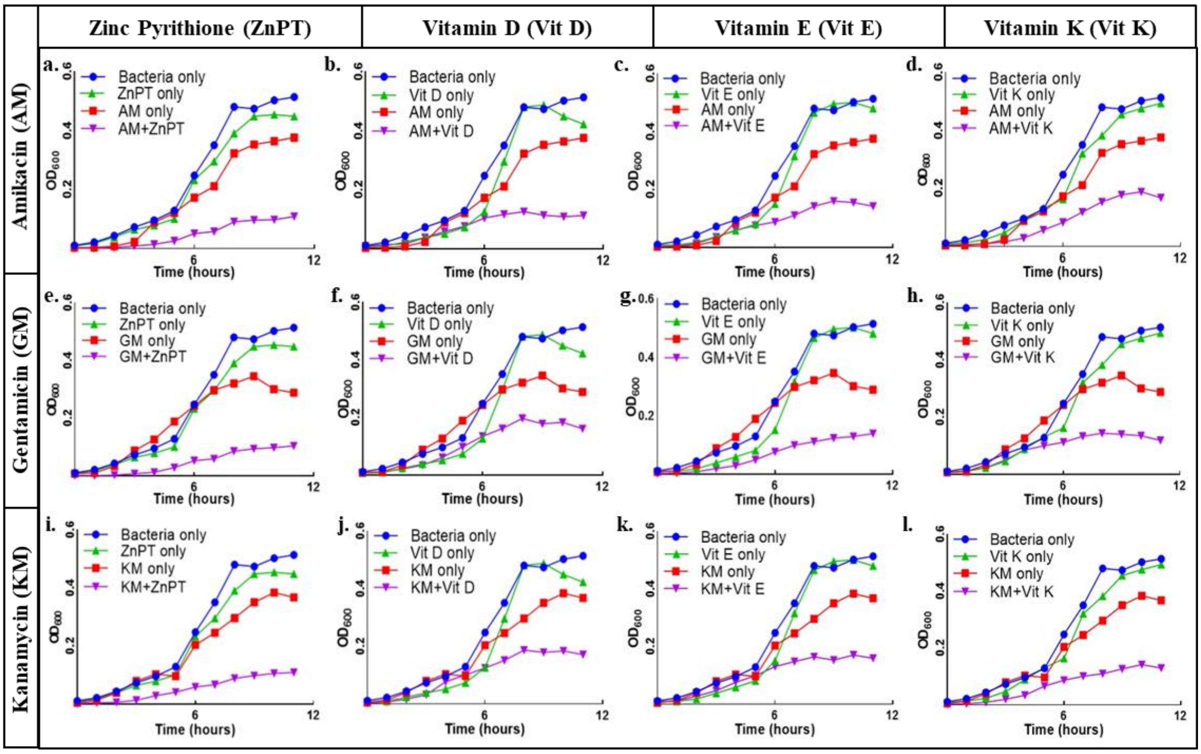

Figure S4: Effects of antibiotic-adjuvants on *Klebsiella pneumoniae* by growth curve analysis. *K. pneumoniae*was found to be resistant to several aminoglycoside antibiotics (AM, KM, GM) but killed by their combination with ZnPT, vitamin D, vitamin E and vitamin K (panel **a-l**). *K. pneumoniae* was cultured in Mueller-Hinton broth at 37^{0}C, with the additional compound indicated in the figure inset and the OD_{600} was observed periodically. In each case, bacterial growth was significantly inhibited by antibiotic when adjuvant was present.

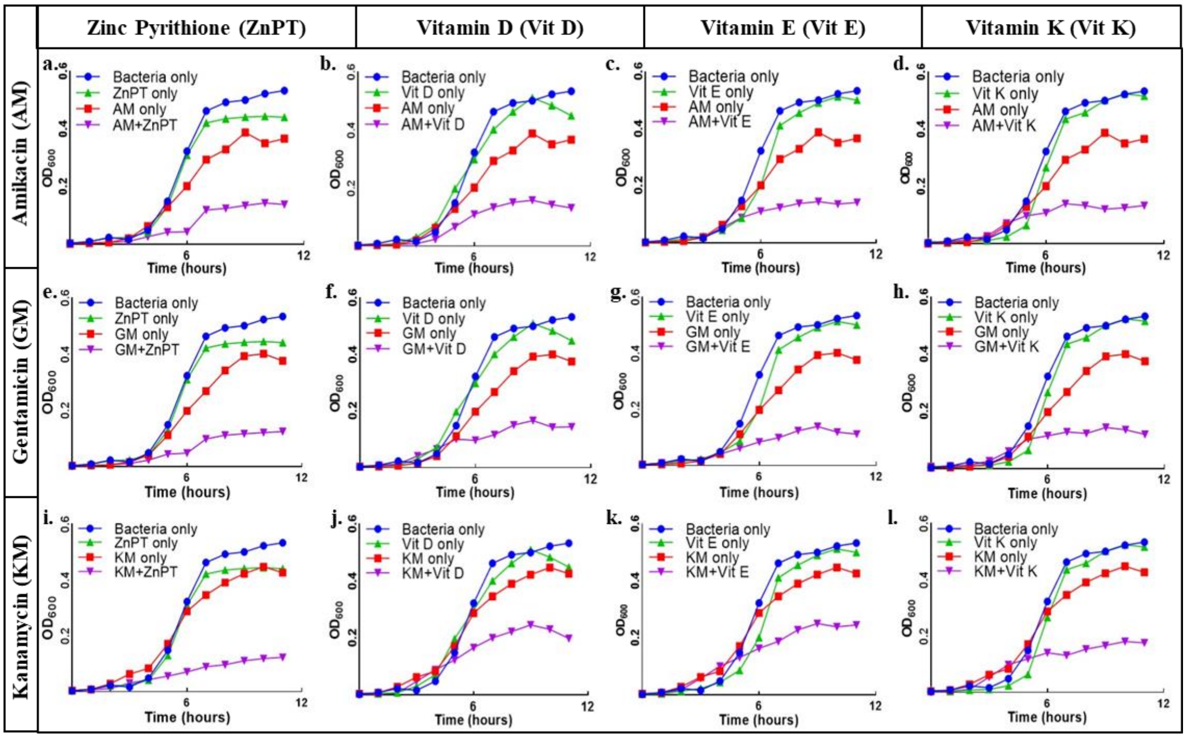

Figure S5: Effects of antibiotic-adjuvants on *Shigella sonnei* by growth curve analysis. *S. sonnei* was found to be resistant to several aminoglycoside antibiotics (AM, KM, GM) but killed by their combination with ZnPT, vitamin D, vitamin E and vitamin K (panel **a-l**). *S. sonnei* was cultured in Mueller-Hinton broth at 37^0C, with the additional compound indicated in the figure inset and the OD_{600} was observed periodically. In each case, bacterial growth was significantly inhibited by antibiotic when adjuvant was present.

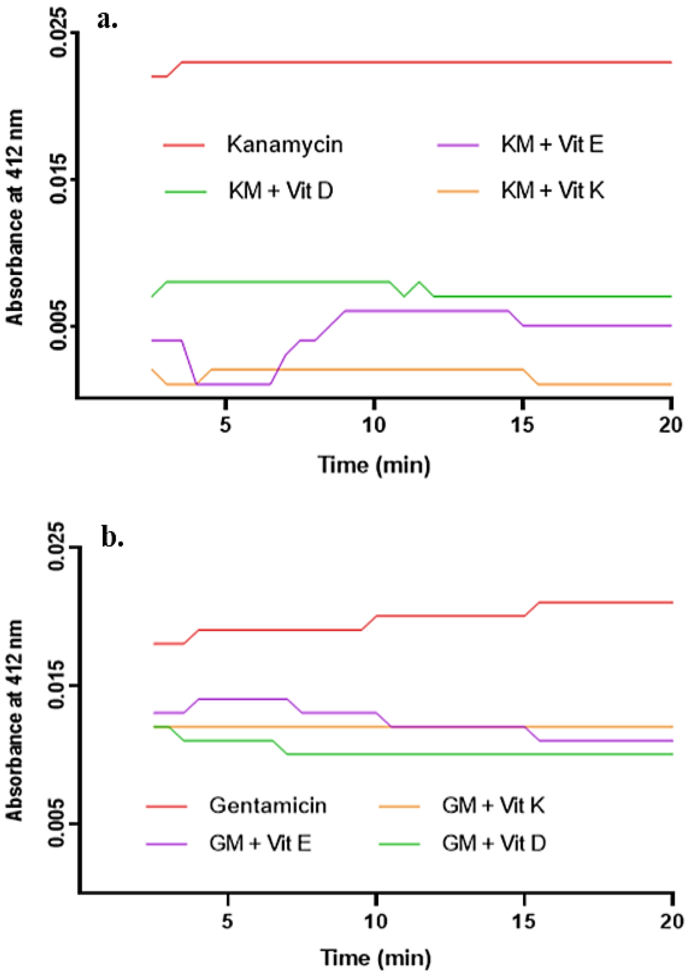

Figure S6 (I): Comparative analysis of AAC(6′)-Ib activity in the presence of substrates kanamycin (a), gentamicin (b) and antibiotic-adjuvant combinations. In presence of the antibiotic, the enzyme showed the highest activity by transferring acetyl group from natural ligand acetyl CoA to antibiotic, whereas the combination of the antibiotics and adjuvants (vitamin D, E and K) significantly reduced the activity of AAC(6′)-Ib.

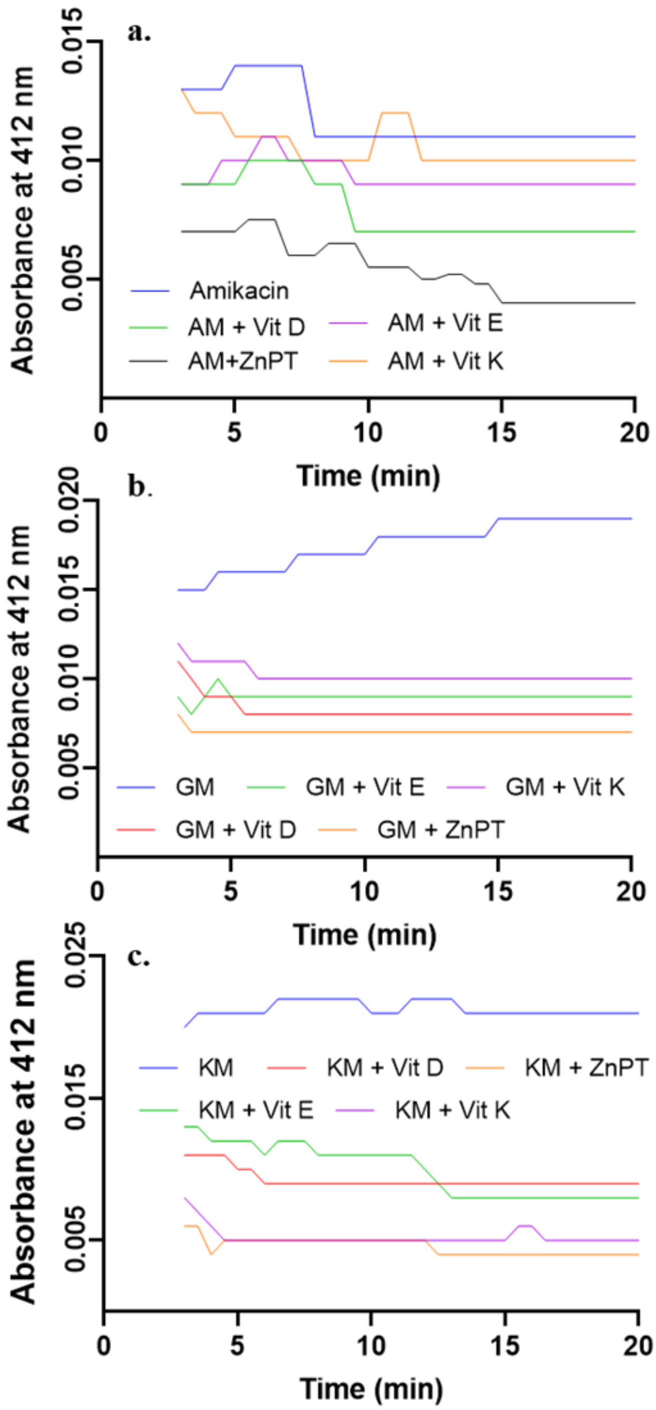

Figure S6 (II): Comparative analysis of AAC(6′)-Ib activity of *K. pneumoniae* in the presence of substrates amikacin (a), kanamycin (b), gentamicin (c) and antibiotic-adjuvant combinations. In presence of the antibiotic, the enzyme showed the highest activity by transferring acetyl group from natural ligand acetyl CoA to antibiotic, whereas the combination of the antibiotics and adjuvants (ZnPT, vitamin D, E and K) significantly reduced the activity of AAC(6′)-Ib.

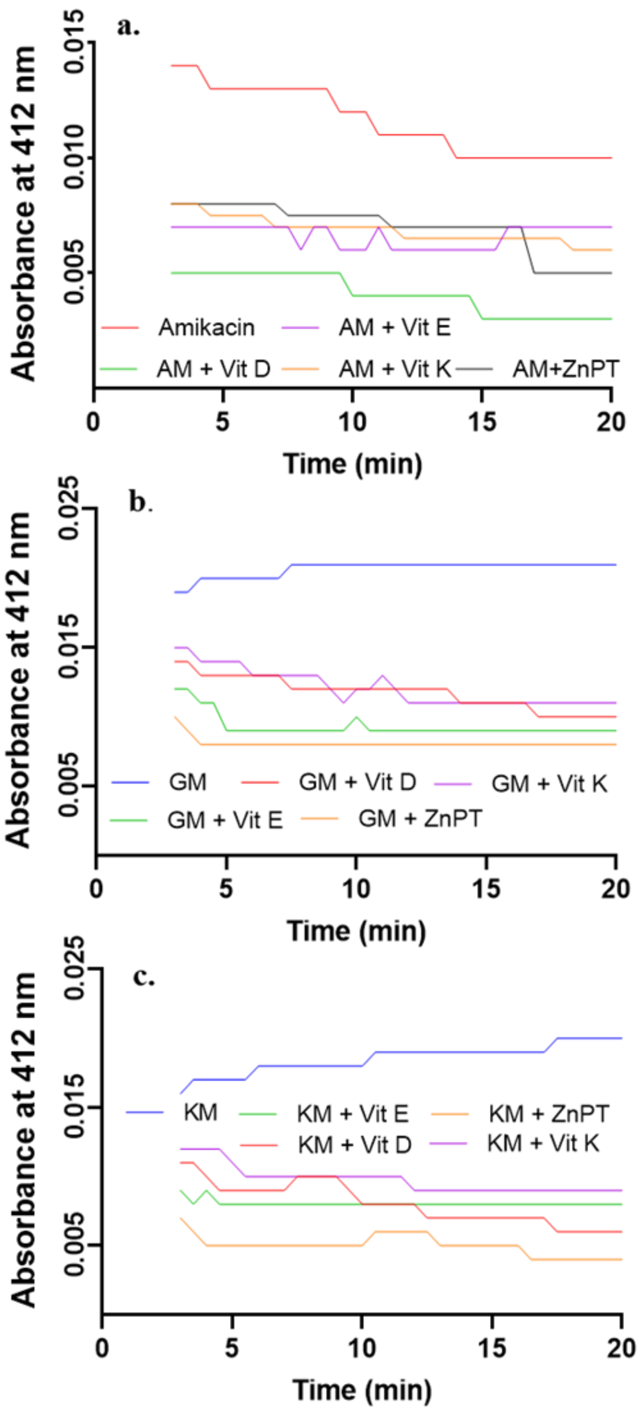

Figure S6 (III): Comparative analysis of AAC(6′)-Ib activity of *S. sonnei* in the presence of substrates amikacin (a), kanamycin (b) and gentamicin (c) and antibiotic-adjuvant combinations. In presence of the antibiotic, the enzyme showed the highest activity by transferring acetyl group from natural ligand acetyl CoA to antibiotic, whereas the combination of the antibiotics and adjuvants (ZnPT, vitamin D, E and K) significantly reduced the activity of AAC(6′)-Ib.

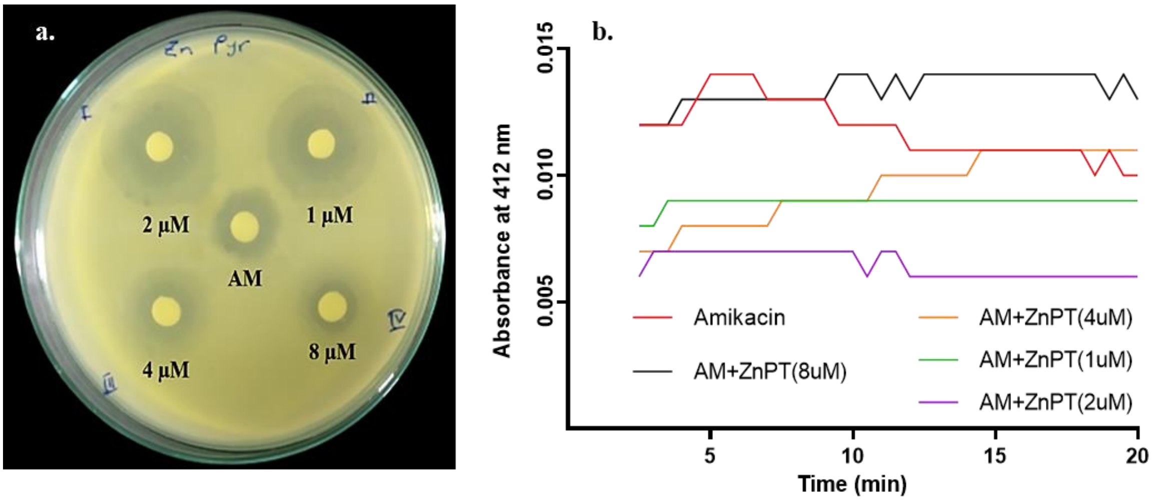

Figure S7: Optimization of ZnPT concentration by antibiotic sensitivity test (a) and AAC(6′)-Ib activity assay (b). In both cases, ZnPT concentration of 2 µM caused the highest growth inhibition and maximum reduction of enzyme activity compared to 1, 4 and 8 µM ZnPT.

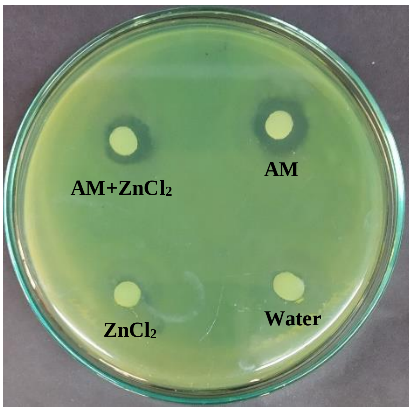

Figure S8: Effect of $ZnCl_2$ on retention of amikacin (AM) activity. Zone of inhibition produced by both amikacin and amikacin and zinc chloride combination against *E. coli* was found to be similar which suggests no role of zinc chloride on amikacin activity.

Supplementary Text 1:

E.coli: Ser-92, Tyr-93, Val-94, Ala-95, Gly101, Arg-102, Trp-103, Glu-104, Glu-105, Glu-106, Thr-107, Gly-129, Thr-130, Leu-136, Val-137, Leu-139, Leu-140, Phe-141, Asp-143

K.pneumoniae**:** Gln-93, Ser-94, Tyr-95, Val-96, Ala-97, Gly-103, Trp-104, Trp-105, Glu-106, Glu-107, Glu-108, Thr-109, Leu-130, Gly-131, Thr-132, Leu-138, Val-139, Leu-141, Leu-142, Phe-143, Asp-145

S. sonnei**:** Leu-25, Ser-28, Pro-94, Gly-95, Val-96, Arg-97, Gly-98, Ala-104, Asn-105, Ala-106, Ser-107, Gln-108, Leu-109, Gly-110, Val-131, Thr-132, Lys-133, Ser-139, Pro-140, Asn-142, Leu-143, Arg-144, Ile-146.
